# SARS-CoV-2 infection of African green monkeys results in mild respiratory disease discernible by PET/CT imaging and shedding of infectious virus from both respiratory and gastrointestinal tracts

**DOI:** 10.1371/journal.ppat.1008903

**Published:** 2020-09-18

**Authors:** Amy L. Hartman, Sham Nambulli, Cynthia M. McMillen, Alexander G. White, Natasha Louise Tilston-Lunel, Joseph R. Albe, Emily Cottle, Matthew D. Dunn, L. James Frye, Theron H. Gilliland, Emily L. Olsen, Katherine J. O’Malley, Madeline M. Schwarz, Jaime A. Tomko, Reagan C. Walker, Mengying Xia, Matthew S. Hartman, Edwin Klein, Charles A. Scanga, JoAnne L. Flynn, William B. Klimstra, Anita K. McElroy, Douglas S. Reed, W. Paul Duprex

**Affiliations:** 1 Center for Vaccine Research, School of Medicine, University of Pittsburgh, Pittsburgh, Pennsylvania, United States of America; 2 Department of Infectious Diseases and Microbiology, School of Public Health, University of Pittsburgh, Pittsburgh, Pennsylvania, United States of America; 3 Department of Microbiology and Molecular Genetics, School of Medicine, University of Pittsburgh, Pittsburgh, Pennsylvania, United States of America; 4 Division of Laboratory Animal Resources, University of Pittsburgh, Pittsburgh, Pennsylvania, United States of America; 5 Department of Radiology, Allegheny Health Network, Pittsburgh, Pennsylvania, United States of America; 6 Department of Immunology, School of Medicine, University of Pittsburgh, Pittsburgh, Pennsylvania, United States of America; 7 Department of Pediatrics, Division of Pediatric Infectious Disease, University of Pittsburgh, Pittsburgh, Pennsylvania, United States of America; Johns Hopkins University Bloomberg School of Public Health, UNITED STATES

## Abstract

Vaccines are urgently needed to combat the global coronavirus disease 2019 (COVID-19) pandemic, and testing of candidate vaccines in an appropriate non-human primate (NHP) model is a critical step in the process. Infection of African green monkeys (AGM) with a low passage human isolate of SARS-CoV-2 by aerosol or mucosal exposure resulted in mild clinical infection with a transient decrease in lung tidal volume. Imaging with human clinical-grade ^18^F-fluoro-2-deoxy-D-glucose positron emission tomography (^18^F-FDG PET) co-registered with computed tomography (CT) revealed pulmonary lesions at 4 days post-infection (dpi) that resolved over time. Infectious virus was shed from both respiratory and gastrointestinal (GI) tracts in all animals in a biphasic manner, first between 2–7 dpi followed by a recrudescence at 14–21 dpi. Viral RNA (vRNA) was found throughout both respiratory and gastrointestinal systems at necropsy with higher levels of vRNA found within the GI tract tissues. All animals seroconverted simultaneously for IgM and IgG, which has also been documented in human COVID-19 cases. Young AGM represent an species to study mild/subclinical COVID-19 disease and with possible insights into live virus shedding. Future vaccine evaluation can be performed in AGM with correlates of efficacy being lung lesions by PET/CT, virus shedding, and tissue viral load.

## Author summary

SARS-CoV-2 infection can result in asymptomatic, mild or severe disease in humans. Vaccines against SARS-CoV-2 are paramount for combating COVID-19 disease and curtailing the pandemic. Large animal models are critical for early vaccine testing before human clinical trials. Here, we found that infection of African green monkeys (AGM) with SARS-CoV-2 resulted in mild disease yet lesions were detectable by PET/CT imaging of the lungs. Shedding of infectious virus from both respiratory and gastrointestinal tracts was also documented. This study provides a detailed account of the pathogenesis of a low-passage SARS-CoV-2 isolate in the AGM model and suggests that AGM can be used for preclinical evaluation of candidate vaccines and therapeutic interventions.

## Introduction

The unprecedented and rapidly spreading coronavirus disease 2019 (COVID-19) pandemic caused by the emerging coronavirus SARS-CoV-2 calls for swift testing of vaccine candidates prior to initiation of human clinical trials. Safety must be prioritized over speed when considering a vaccine that will likely be administered to hundreds of millions of people. Non-human primates (NHPs) serve a unique and important purpose for pre-clinical testing of candidate human vaccines, given their close genetic relatedness with humans. As such, studies in NHPs are geared towards identifying the most appropriate species that recapitulates human disease. Comprehensive dissection of the longitudinal viral pathogenesis in NHP models is critical for successful evaluation of the efficacy of vaccines, antibody therapeutics, and small molecules in pre-clinical studies.

Several NHP models have been reported with SARS-CoV-2 using rhesus and/or cynomolgus macaques and African green monkeys (AGMs) [1–4]. While used less frequently in biomedical research than rhesus or cynomolgus macaques, AGMs are an old-world NHP species and a natural host for simian immunodeficiency virus (SIV) [5]. In addition to SIV, AGMs have been used as models for pulmonary infectious diseases such as Rift Valley fever, pneumonic plague, human parainfluenza virus, SARS-CoV-1, and Nipah virus [6–10]. This study was designed to understand the pathogenesis of human isolates of SARS-CoV-2 in AGM, including real-time dynamics of virus shedding and whether clinical-grade imaging of NHPs can be used to detect subclinical infections.

Young adult male AGMs were infected with a low-passage clinical isolate of SARS-CoV-2 and comprehensive longitudinal disease parameters were compared after either small particle aerosol or multi-route mucosal/intratracheal infection. All AGMs developed mild disease regardless of the route of exposure or infectious dose. Pulmonary lesions were detectable by PET/CT in the acute phase and subsequently resolved. All AGMs exhibited prolonged shedding of infectious virus from oral, nasal, conjunctival, and rectal mucosal surfaces. Viral RNA (vRNA) remained detectable throughout both the respiratory and GI tissues at necropsy in the absence of replication-competent virus. These results show multiple routes are involved in viral shedding and that shedding occurs over a protracted period of time in subclinical animals, both of which provide insight into potential SARS-CoV-2 transmission from COVID-19 patients.

## Results

### Infection of young AGMs with SARS-CoV-2 resulted in mild respiratory disease

Healthy male AGMs (~3.5 years of age) were infected with passage 3 (P3) of a human isolate of SARS-CoV-2 from Munich, Germany (S1 Table) [11]. As described [11], this isolate contains the spike protein D614G mutation. Four animals were infected using a small particle aerosol (designated A1-A4) and two were infected by a multi-route mucosal exposure (designated M1, M2) involving administration of virus into the oral, nasal, and ocular mucosal surfaces and intratracheal instillation using a bronchoscope. Aerosol inhaled exposure doses ranged from 3.7–4.2 log_10_ pfu of virus due to the standard ~2 log loss of virus after nebulization. Multi-route mucosal exposures were 6.4 log_10_ pfu. After infection, animals were anesthetized for blood draws, mucosal swabs, plethysmography, and chest radiography at regular intervals post-infection.

Clinical disease was mild in all six animals (S1A Fig). Respiratory function revealed a transient decrease in the volume of air inhaled, or tidal volume, at 7 dpi (S1B Fig), while other parameters including frequency and expiratory time were within normal limits. In the two mucosally infected AGM and in one aerosol infected AGM, there were several short spikes of fever (maximum deviation 2.4–3.4°C) during the course of infection (S2 Fig). For these three animals, the average significant elevation in temperature was less than 1°C for either route of infection, suggesting an overall low-grade fever. The other three aerosol-infected AGMs, A1-A3, developed mild hypothermia response around 5–7 dpi that persisted for most of the remainder of the study (S2 Fig). Complete blood counts (CBC) revealed a transient decrease in lymphocytes and platelets and an increase in neutrophils (S1 Fig); this is also seen in human COVID-19 patients [12, 13]. Blood chemistry analysis demonstrated decreases in amylase and blood urea nitrogen (BUN) and no elevation in liver enzyme levels (S3 Fig).

### SARS-CoV-2 infected AGMs shed infectious virus from respiratory and gastrointestinal tracts

**Fig 1:**
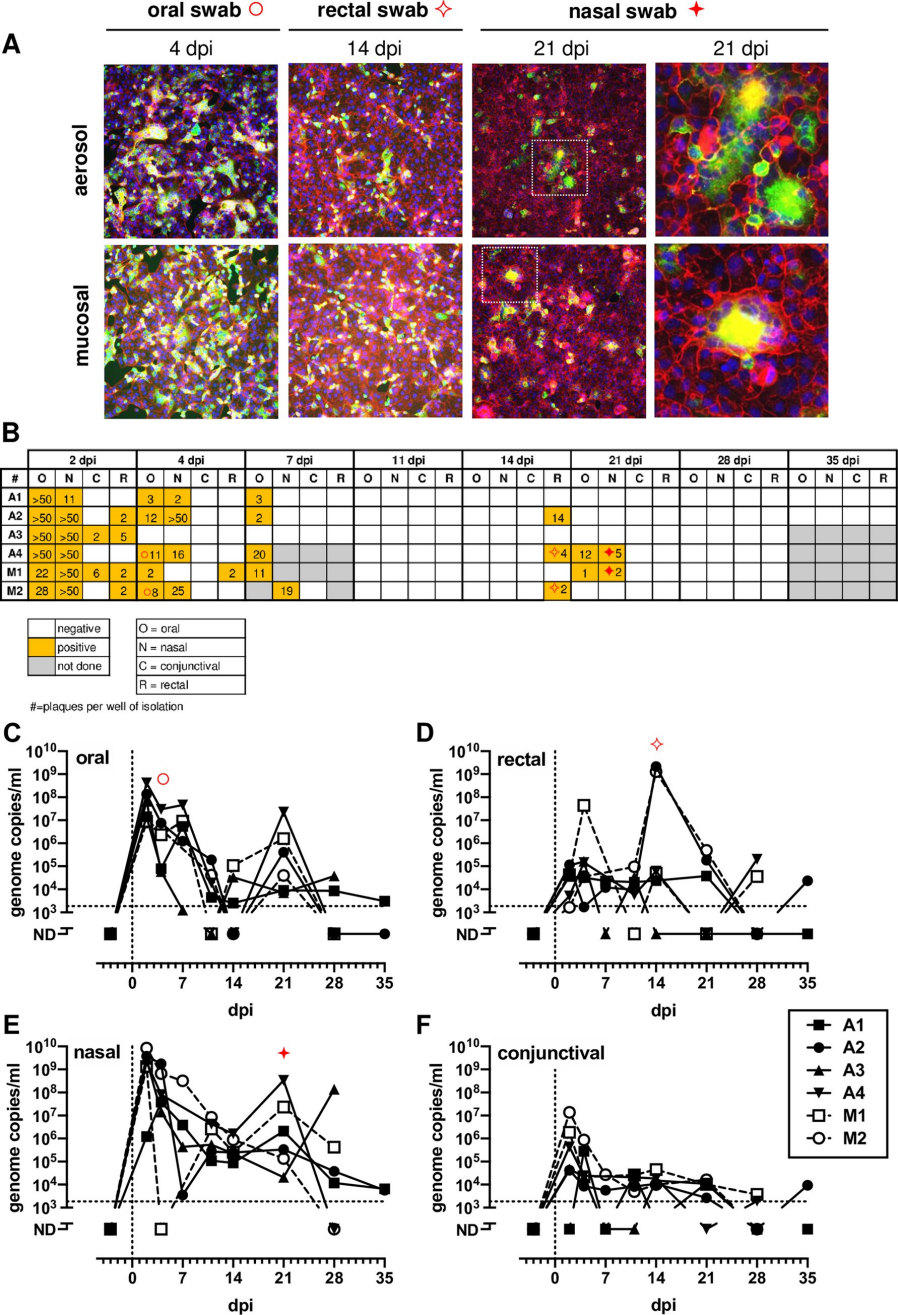
Detection and isolation of SARS-CoV-2 in swabs. (A) Representative virus isolations confirmed by immunofluorescence using anti-SARS2 spike Ab with phalloidin as background. (B) Isolation results from swabs. Number in each square represents the # of plaques obtained from isolation. (C-F) vRNA in swabs measured by q-RT-PCR. AGM infected by aerosol (closed symbols/solid lines; n = 4) or multi-route mucosal (open symbols/dashed lines; n = 2). In B-F, Red symbols highlight representative IFA images shown in (A).

Virus isolations and q-RT-PCR analysis were performed for oral, rectal, nasal, and conjunctival swab samples obtained over the course of the infection. SARS-CoV-2 isolations were confirmed using indirect immunofluorescence using an anti-spike antibody (Fig 1A). Syncytia were present in *in vitro* isolations, particularly at the later time points (e.g. 21 dpi). Replicating virus was detected in all nasal and oral swabs from all animals on 2 and 4 dpi (Fig 1B). On 2 dpi, 5/6 rectal swabs and 2/6 ocular swabs were positive for infectious virus. All swabs were broadly negative from all animals at 11 dpi. However, there was a resurgence in the presence of replication competent virus on 14 and 21 dpi in samples from the respiratory and GI tracts (Fig 1A and 1B). q-RT-PCR results confirmed the virus isolation results, with a peak in vRNA detection in oral, nasal and conjunctival swabs between 2–7 dpi and a second spike in rectal samples 21 dpi (Fig 1C–1F). There was consistent detection of vRNA through 28 dpi although at this stage, infectious virus was not isolated from either swabs or necropsy tissues. Three of the swab isolates were sequenced, and the furin cleavage site and D614G mutation were maintained as in the original inoculum (S4 Fig) [11].

### Pulmonary infection in SARS-CoV-2 infected AGM was detected by PET/CT imaging

Molecular imaging using positron emission tomography (PET) with the radiotracer ^18^F-FDG provides a sensitive measurement of metabolic activity within specific anatomical compartments. When co-registered with computed tomography (CT), PET/CT can provide detailed anatomic structure overlaid with areas of high metabolic activity revealed by the tracer. FDG-based PET/CT has been used in NHPs for measurement of lung granulomas caused by infection with *Mycobacteria tuberculosis* [14–19]. To determine whether lung infection with SARS-CoV-2 could be visualized using FDG-mediated PET/CT, imaging was performed pre-infection, 4 dpi, and 11 dpi for 5/6 animals and also on 18 dpi for 1 animal. To quantify overall disease burden in the lungs at the various time points, a disease-associated total lung FDG activity level was calculated to measure the total lung inflammation. Analysis of the maximum SUVs in thoracic lymph nodes (LNs) was also conducted.

**Fig 2:**
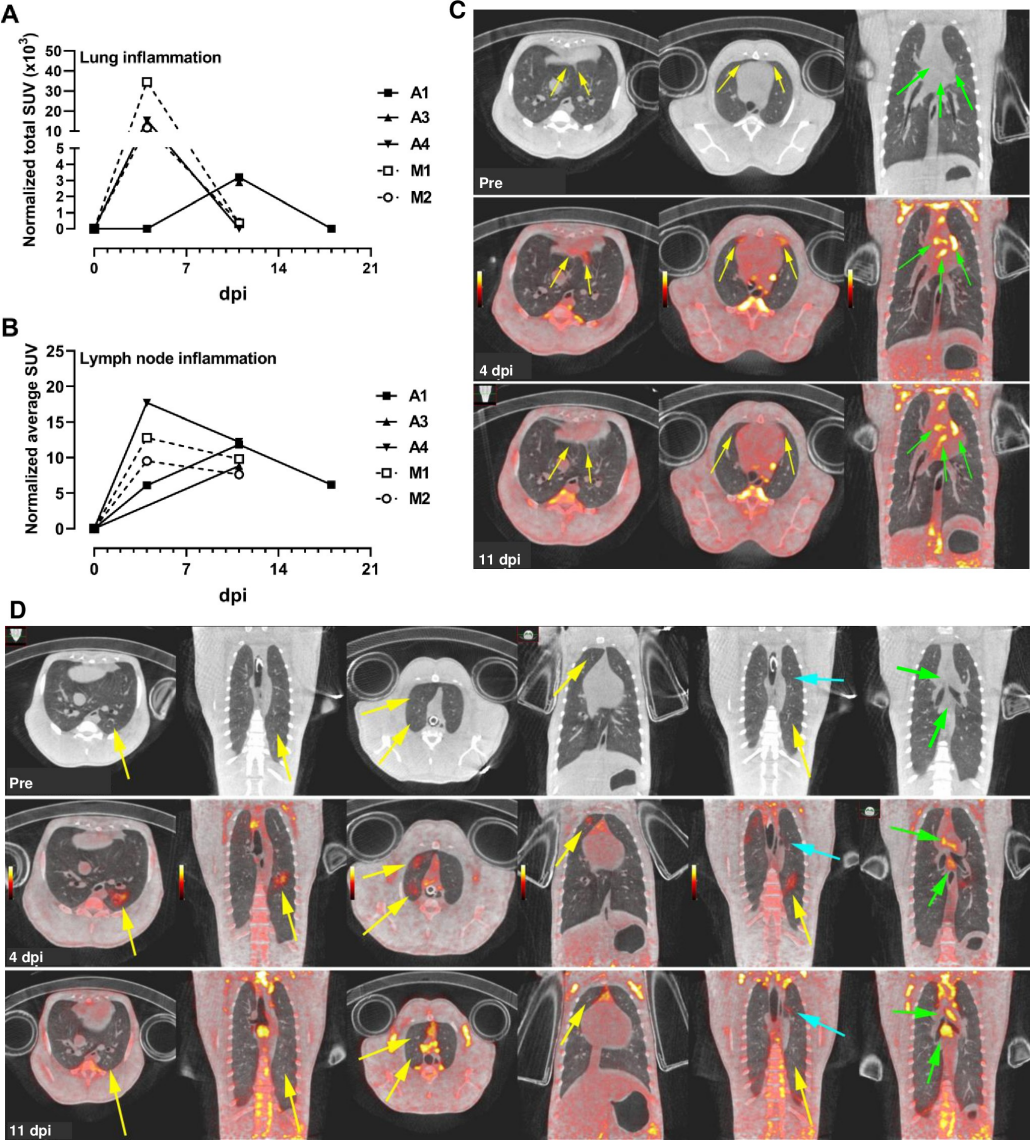
PET/CT imaging of SARS-CoV-2-infected AGM. (A) and (B) Region-of-interest analysis on PET images of animals infected with SARS-CoV-2. (A) Measurement of total lung inflammation via FDG uptake over time. (B) Measurement of average lymph node inflammation over time. Aerosol (closed symbols/solid lines; n = 3); multi-route mucosal (open symbols/dashed lines; n = 2). Animal A2 is not included in (A) and (B) because only CT scans were performed. (C) For AGM A4 (aerosol), only CT scan was obtained pre-infection; PET/CT at was obtained at 4 dpi and 11 dpi. (D) For AGM M1 (multi-route mucosal), only CT scan was obtained pre-infection; PET/CT at was obtained at 4 dpi and 11 dpi.. Pulmonary infection (yellow arrows); thoracic lymph nodes (green arrows). Cyan arrow highlights new focal area of disease visible on 11 dpi. PET color scale is from 0–15 SUV.

Both mucosally-infected animals and one of the aerosol animals (A4) had significant lung inflammation at 4 dpi based on FDG uptake; these lesions resolved by 11 dpi (Fig 2A). The LNs in these three animals also showed the highest FDG uptake at 4 dpi, while all 6 AGM had substantial FDG uptake in the LNs at either 4 or 11 dpi (Fig 2B). Overall, the extent of disease visualized within the lungs of infected AGMs was modest at all the time points examined (S5 and S6 Figs). At 4 dpi, aerosol-infected animal A4 developed areas of disease with a pneumonia appearance in the anterior portions of the accessory lobe and left lower lobe as well as pleural ground glass opacities in the anterior portions of the right and left middle lobes (Fig 2C). These lesions were resolving by 11 dpi. Peak uptake in the thoracic lymph nodes of this animal was seen at 4 dpi (Fig 2B). AGM M1 was infected via mucosal exposure and had the most extensive lung disease of all AGMs based on PET imaging. Lesions consisted of diffuse ground glass opacities with corresponding high FDG uptake in the left lower and right upper lobes (Fig 2D). These areas resolved by day 11, but a new focal area of disease was present that day (cyan arrow; Fig 2D). Chest radiographs were taken on all longitudinal sampling days. Only animal M1 had detectable radiographic abnormalities with mild non-specific infiltrates present in the left lower lobe at 2 and 4 dpi that were resolving by 7 dpi (S7 Fig).

### Synchronous seroconversion in SARS-CoV-2-infected AGM

**Fig 3:**
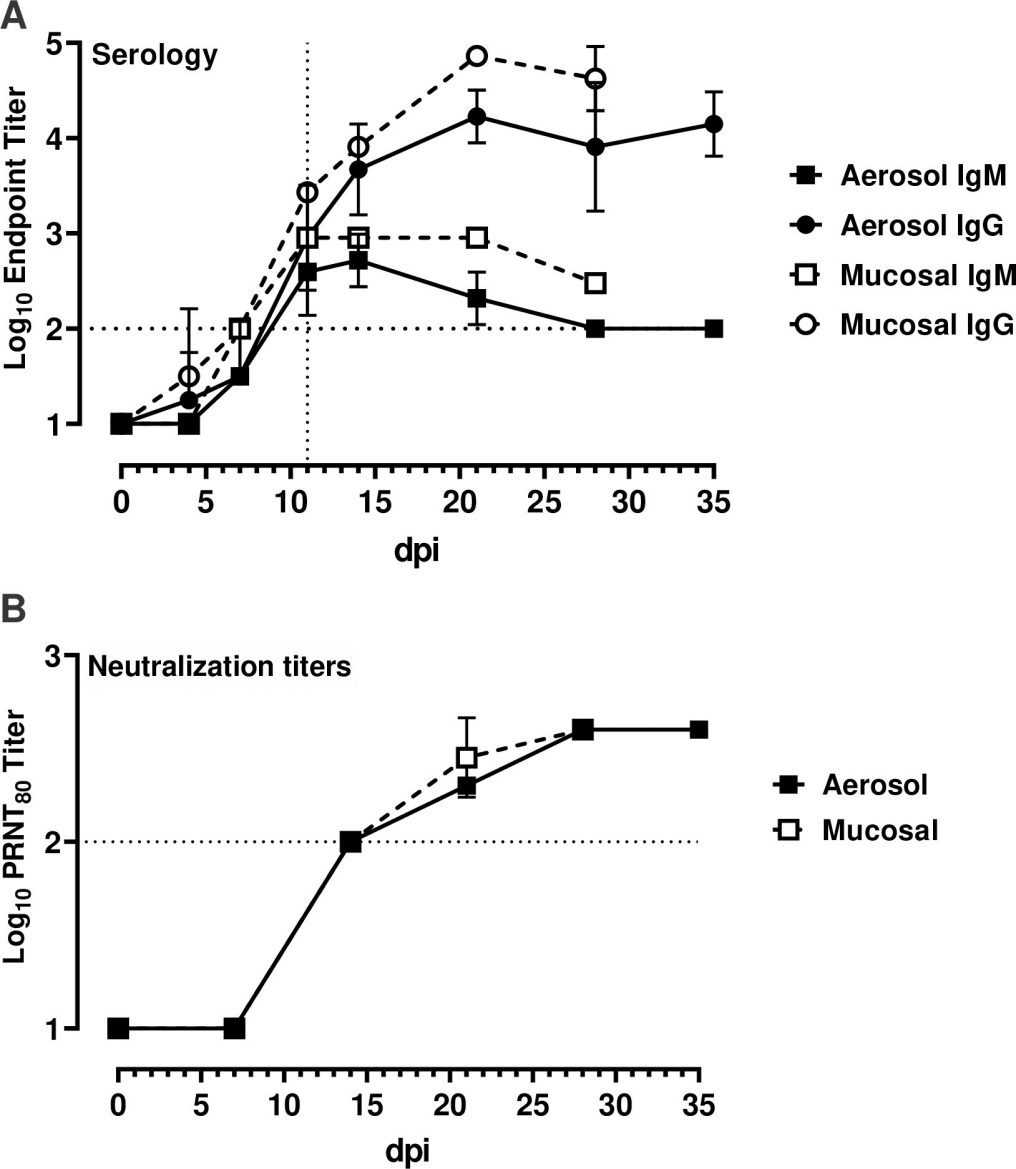
Seroconversion and neutralization in SARS-CoV-2 infected AGMs. Serial plasma samples were assayed for virus specific antibodies or neutralization capacity. (A) virus-specific IgG and IgM were measured using a SARS-CoV-2 spike receptor binding domain-based ELISA. (B) neutralization titers were determined by a plaque-reduction neutralization 80% assay (PRNT_80_). The horizontal dotted line represents the limit of detection of each assay.

In order to evaluate the kinetics of seroconversion, serial plasma samples from each SARS-CoV-2 infected AGM were assayed for antibodies against the SARS-CoV-2 spike protein receptor binding domain (RBD) using an indirect ELISA (Fig 3A). All animals generated both IgM and IgG antibodies against SARS-CoV-2. Notably, there was simultaneous seroconversion of both IgM and IgG. This contradicts the classic immunology paradigm of IgM proceeding IgG in response to antigen exposure, however this mirrors precisely what occurs in COVID-19 patients [20–23], further validating the utility of this AGM model. All animals seroconverted in the second week post exposure and those that were exposed via the mucosal route had higher overall titers than those that received the virus via the aerosol route. This could reflect the fact that mucosal infected animals received a higher challenge dose than aerosol infected animals. Neutralization of SARS-CoV-2 was measured in matched samples using a plaque-reduction neutralization-80% test (PRNT_80_) (Fig 3B). Neutralizing antibodies were present in samples collected 7–11 dpi. There were no differences in kinetics or activity between the two exposure routes demonstrating that higher dose received during multi-route mucosal infection neither affected the onset nor strength of the humoral immune response.

### Cytokinemia and immune activation over time revealed early response to viral infection

**Fig 4:**
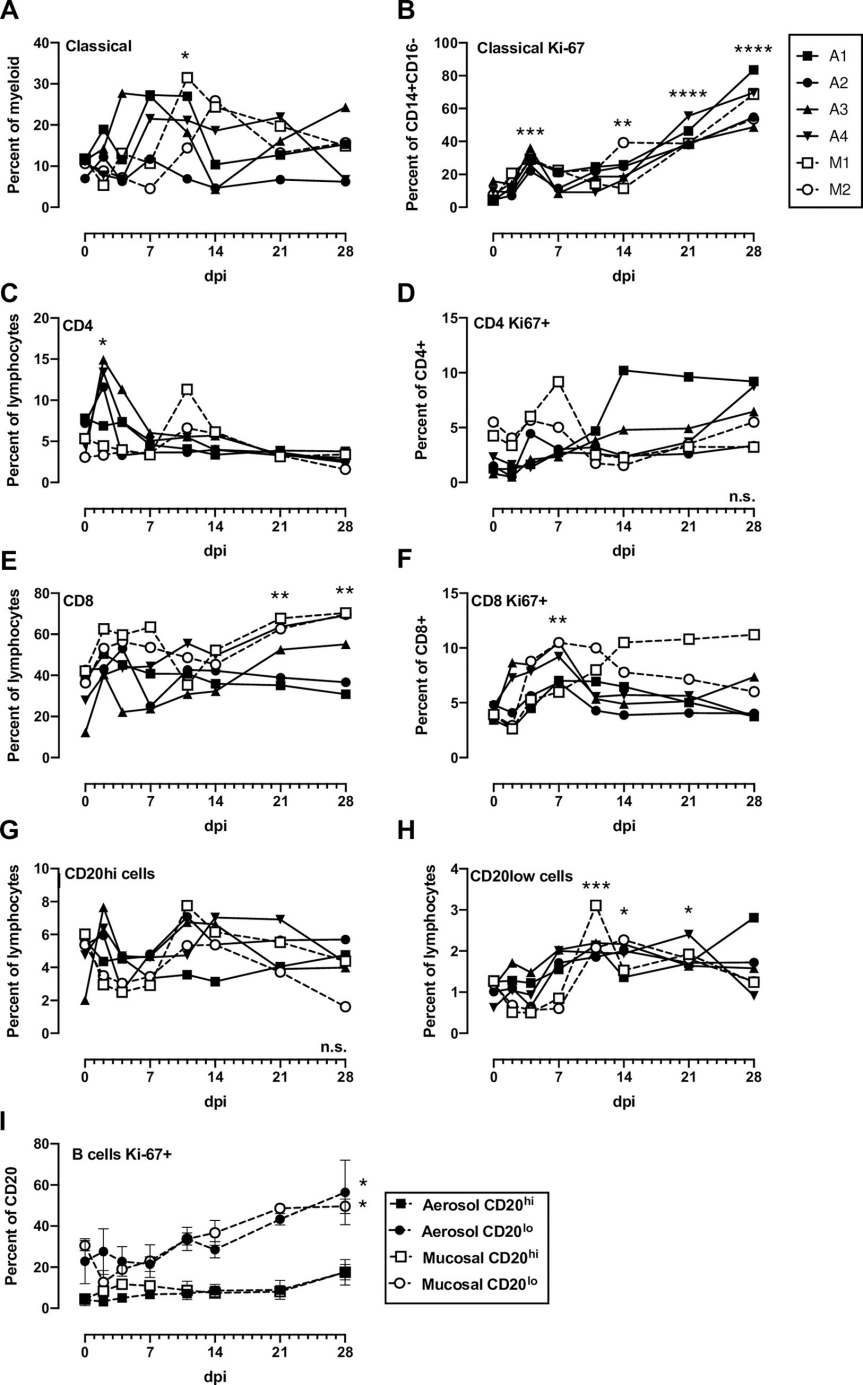
Blood cell populations in SARS-CoV-2 infected AGMs. (A) Classical monocytes (CD14+CD16-), (B) Ki-16+ classical monocytes, (C) CD4+ T cells, (D) CD4+Ki-67+ cells, (E) CD8+ T cells, (F) CD8+Ki-67+ cells, (G) B cells with high CD20 expression, (H) B cells with low CD20 expression (plasmablasts), (I) Grouped expression of Ki-67 on CD20^hi^ and CD20^lo^ (plasmablast) populations. 2-way ANOVA with multiple comparisons was used to determine statistical significance compared to 0 dpi and is indicated by asterisks above each time point. N.s. in the lower right corner indicates no significant differences.

**Fig 5:**
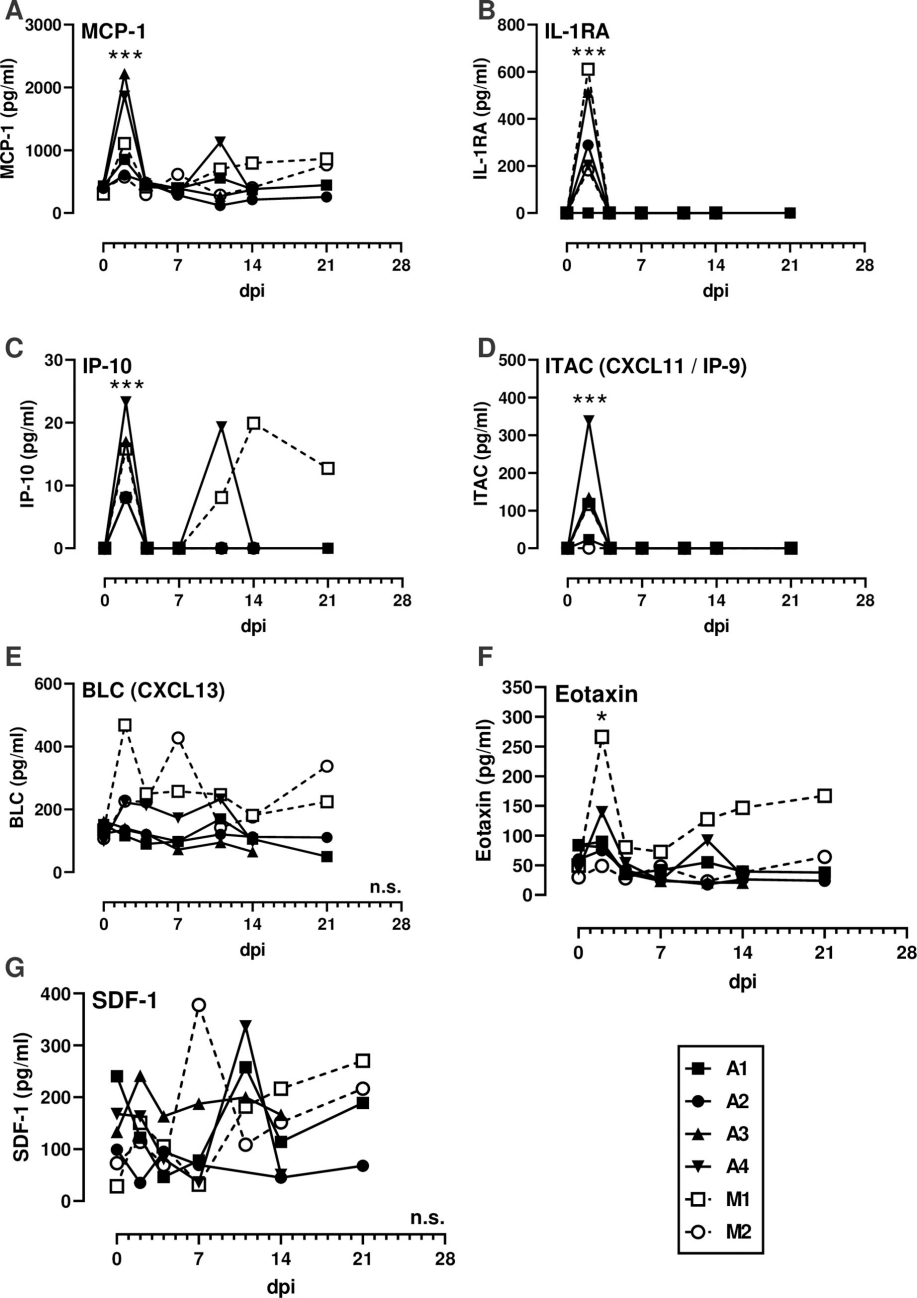
Cytokinemia during SARS-CoV-2 infection. Cytokines were measured in longitudinal plasma samples using the Cytokine & Chemokine 30-Plex NHP ProcartaPlex Panel from Invitrogen. (A) MCP-1; (B) IL-1RA; (C) IP-10; (D) ITAC/CXCL11/IP-9; (E) BLC/CXCL13; (F) Eotaxin; (G) SDF-1. Parameters not shown were below the limit of detection across all animals and time points. Aerosol (closed symbols/solid lines; n = 4); multi-route mucosal (open symbols/dashed lines; n = 2). A 2-way ANOVA with multiple comparisons was used to determine statistical significance compared to 0 dpi and is indicated by asterisks above each time point. N.s. in the lower right corner indicates no significant differences.

PBMC were isolated from whole blood and stained for flow cytometry using either a myeloid or lymphoid panel (S2 Table; S8 and S9 Figs). Classical monocytes (CD14+CD16-) expressing Ki-67 increased after infection in all animals (Fig 4B). The proportions of proliferating (Ki-67+) CD4 and CD8 T cells increased transiently between 2–11 dpi in most animals (Fig 4D–4F). There were sustained increases in proliferating CD8 T cells from mucosally-infected animals (Fig 4D) which is similar to what occurs in human COVID-19 patients [12, 13]. Most notably, an increased frequency of CD20^lo^ B cells was noted in all animals in the second week of infection, accompanied by increased expression of Ki-67 in these cells, which is consisted with a plasmablast phenotype (Fig 4H and 4I). In comparison, CD20^hi^ B cells saw no increase in Ki-67. NK cell frequencies varied over the course of the experiment as did the mean fluorescence intensity (MFI) of CD16 on NK cells with no clear pattern emerging (S10 Fig). This is relevant since COVID-19 patients with severe, but not moderate, disease demonstrated decreased CD16 MFI on NK cells [12]. An NHP-specific multiplex cytokine and chemokine assay was used to measure the levels of 30 analytes in longitudinal plasma samples. The majority of cytokines, including IFN-α, IFN-γ, IL-6, and TNF- α, were undetectable in all animals at all time points tested. For the cytokines that were detectable, most AGMs had early transient peaks in expression of MCP-1, IL-1RA, IP-10, and ITAC at 2 dpi (Fig 5), indicative of a general response to viral infection. Peak plasmablast frequency (Fig 4H) and B lymphocyte chemoattractant (BLC) expression (Fig 5E) coincided with the appearance of virus specific antibodies (Fig 3).

### SARS-CoV-2 vRNA was detected throughout respiratory and gastrointestinal tract at necropsy

**Fig 6:**
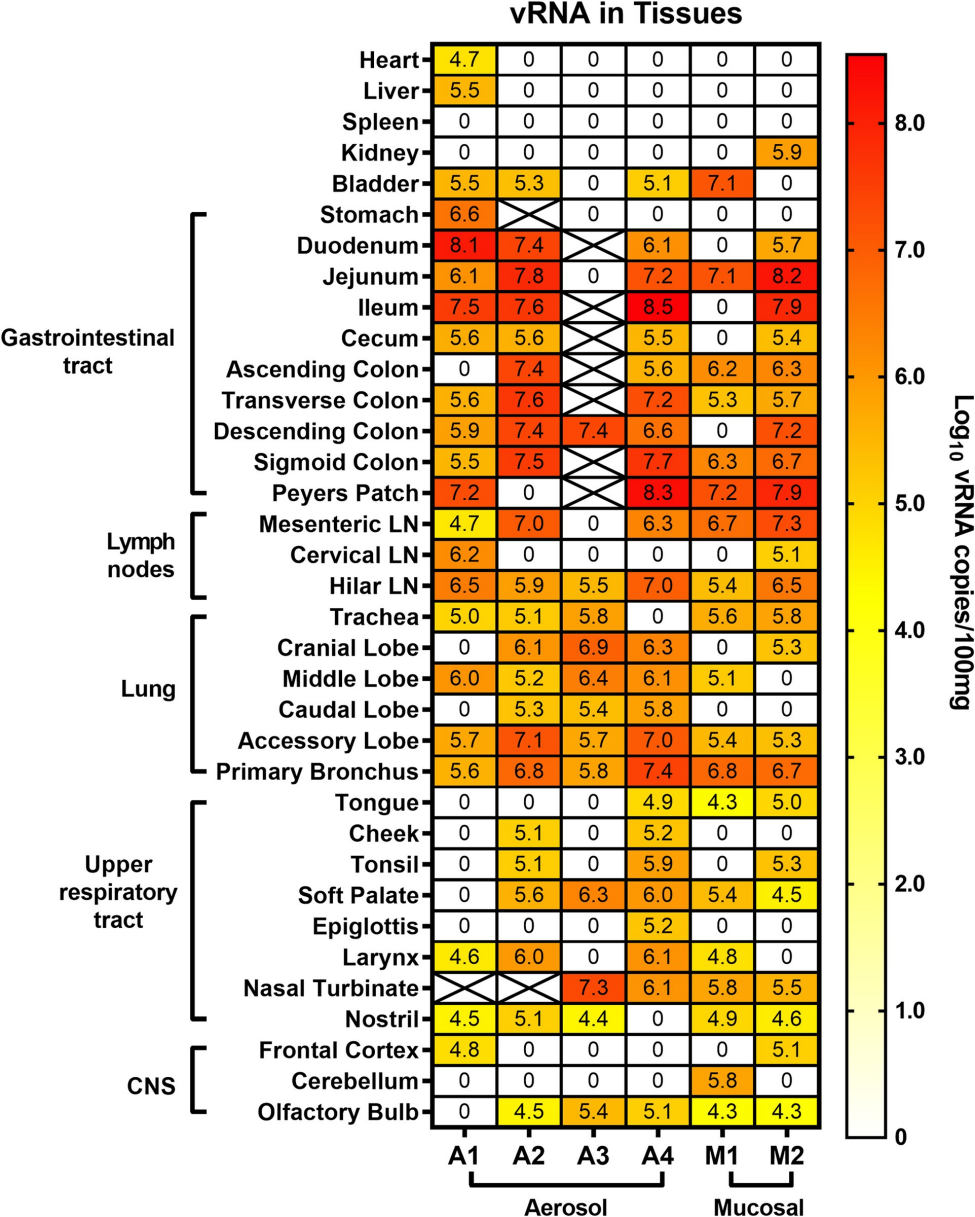
Viral RNA within tissues of SARS-CoV-2 infected AGM. Animals underwent necropsy at 28 dpi (35 dpi for A1 and A2) and the indicated tissues were extracted and tested for viral RNA by q-RT-PCR. Heat map shows the log-transformed vRNA copies per 100 mg of tissue. X indicates the sample was not available or not tested.

Upon euthanasia of each animal at 28 dpi (35 dpi for A1 and A2), full necropsies were performed and tissues were tested by qRT-PCR for levels of vRNA (Fig 6). vRNA was widespread throughout the upper and lower respiratory tracts including the soft palate and nasal turbinates. The overall levels of vRNA detected were comparable between the two exposure routes and doses, indicating that the 100-fold higher infectious dose that the mucosally infected animals received did not translate into higher levels of tissue vRNA at necropsy. More vRNA was detected along the entire gastrointestinal (GI) tract compared to the lower and upper respiratory tracts, highlighting differential tropism of SARS-CoV-2. vRNA was detected in 5/6 olfactory bulbs but was largely absent from the cortex and cerebellum of most animals. The heart, liver, spleen, and kidney were largely devoid of vRNA. Virus isolation attempts on lung and gastrointestinal tissue obtained at necropsy were unsuccessful.

### Residual histopathology detected at necropsy

**Fig 7:**
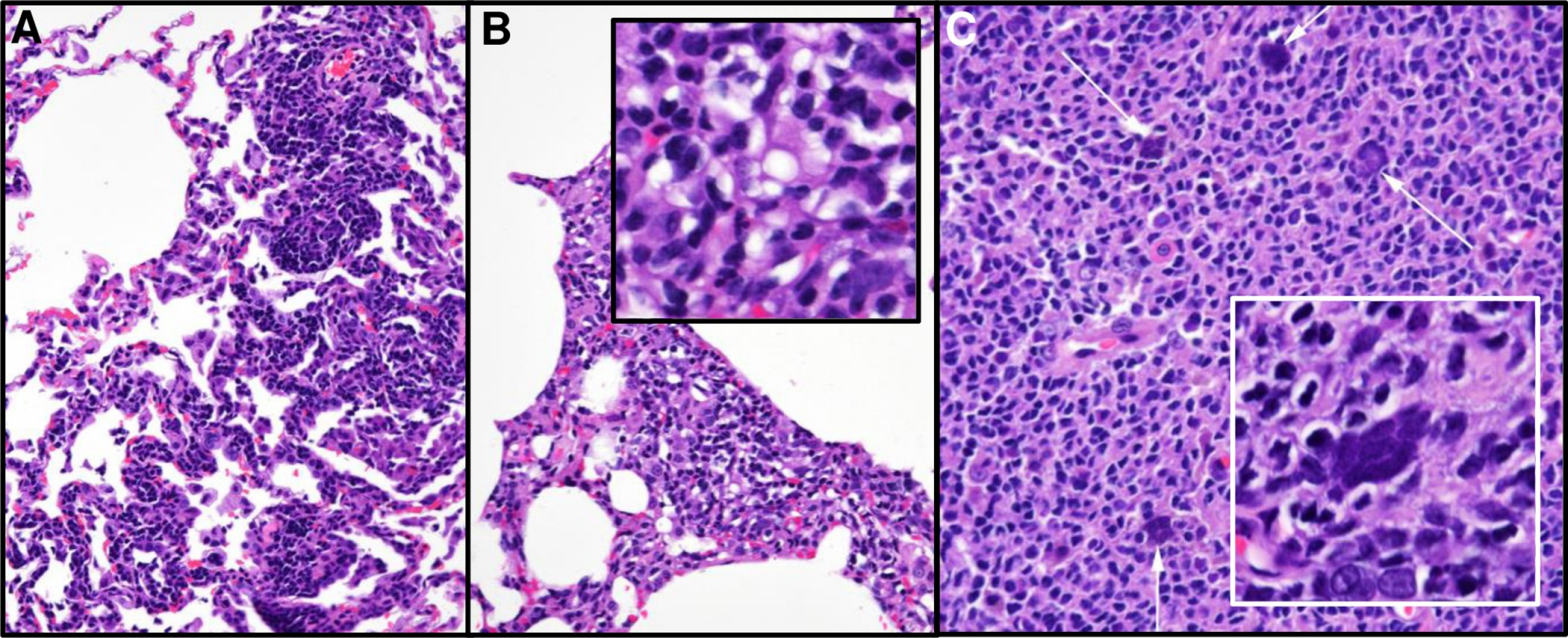
Histopathology detected at necropsy. (A) and (B) Lung (20X) reveals pulmonary foci of mild infiltration and interstitial expansion by lymphocytic and mixed inflammatory cells. (C) Peyer’s patch (20X) shows multiple, syncytialized cells (white arrows). Inset of Peyer’s patch illustrates smudgy syncytia. Histopathology interpreted by a board-certified veterinary pathologist.

Since the AGMs were euthanized at 28 or 35 dpi, tissue specimens for acute histopathology were not obtained. However, lung samples and lymph nodes from animals known to be positive by PET/CT were taken at necropsy and examined by a board-certified veterinary pathologist. Importantly, even 4–5 weeks after infection, several animals demonstrated multiple pulmonary foci of interstitial infiltration and expansion by either lymphocytic or mixed inflammatory infiltrates (Fig 7). While not a specific diagnostic finding, this is a common pathological effect of many pneumotropic viruses and is also consistent with the convalescent stage of a respiratory infection. Interestingly, multiple syncytiated cells were observed in the germinal follicles of Peyer’s patches from two animals (Fig 7B). Given the frequent propensity of coronaviruses, including SARS-CoV-1, to lead to fused cells *in vitro*, our observation that isolated virus from the AGMs form syncytia in Vero-E6 cells (Fig 1A) and the observation of multinucleated syncytial cells in human cases of disease [24, 25], this finding is likely a viral cytopathic effect. Moreover, the Peyer’s patches from 5 out of 6 AGMs contained some of the highest levels of vRNA (Fig 6).

## Discussion

AGMs were used as a non-human primate model for SARS-CoV-1 infection after the 2003 epidemic. Compared to rhesus macaques, AGMs supported enhanced SARS-CoV-1 virus replication and developed more severe lung pathology [10, 26]. They are also an excellent species to model other respiratory diseases such as pneumonic plague and human parainfluenza virus [7, 9]. In this study, COVID-19 disease in young AGMs was mild, reflecting what frequently occurs in young, healthy humans infected with SARS-CoV-2 [27, 28]. Low-grade fever was observed and respiratory symptoms were limited to a transient decrease in tidal volume. Advanced imaging using a metabolic PET probe permitted visualization of pulmonary lesions undetectable by other modalities such as standard chest radiographs. PET/CT is used clinically in managing COVID-19 patients and is effective at visualizing lesions in asymptomatic patients [29–31]. It is notable that the three AGM (A4, M1, M2) that had the most febrile responses also had the highest level of inflammation in the lungs and lymph nodes as detected by PET/CT. PET/CT imaging was also performed in crab-eating macaques infected intra-tracheally with SARS-CoV-2 [32]. Disease in the macaques was mild/subclinical, and PET/CT scans showed areas of pneumonia and consolidation that were FDG avid. The importance of these studies lies in demonstrating that PET/CT imaging can be used to bridge human animal data, particularly with respect to determining reductions in disease burden following vaccination or therapeutic interventions.

A key finding here was that virus isolations followed by immunofluorescence confirmation demonstrated unequivocally that infectious virus was shed from the respiratory and GI early followed by a recrudescence around 14–21 dpi. The majority of *in vivo* SARS-CoV-2 studies use vRNA detection as a surrogate for infectious virus. The biphasic shedding pattern was unexpected and highlights the need to study longer term infection in animal models. vRNA was still detectable at substantial levels throughout the entire GI tract at 4–5 weeks after infection, and GI titers were 10-100-fold higher than what was found throughout the upper and lower respiratory tracts. This demonstrates the tropism of SARS-CoV-2 for intestinal tissues and the importance of GI replication in the viral pathogenesis. Transmission of SARS-CoV-1 through fecal matter contributed to super-spreader events during the 2003 outbreak [33, 34], and the mechanism underlying this remains largely unexplored. For SARS-CoV-2, vRNA and infectious virus has been detected in feces of COVID-19 patients with or without GI symptoms [35–37]. The AGM model will allow an understanding of how SARS-CoV-2 is shed from the GI tract and potentially transmitted via feces.

In a recent study with cynomolgus macaques, SARS-CoV-2 infection was mild to subclinical even in aged animals, shedding of vRNA from mucosal surfaces was limited to early time points, and vRNA at necropsy was largely limited to respiratory tissues [3]. A preprint study in rhesus macaques showed transient clinical disease marked by brief fever right after infection, transient weight loss, mild respiratory depression, and pulmonary infiltrates by chest radiograph [2]. Upon necropsy, vRNA was found primarily in the lungs and upper respiratory tract and not in the GI tract. Another preprint study in rhesus macaques found similar limited disease with shedding of vRNA and infiltrates by chest radiograph [4].

The current consensus regardless of NHP species or viral isolates is that infection with SARS-CoV-2 leads to mild or subclinical infection, with shedding of virus from the respiratory tract. Severe disease or lethality has been rarely seen after either mucosal or aerosol exposure. One distinguishing feature of the AGM model appears to be tropism of virus for tissues throughout the entire gastrointestinal tract, as we found here, including substantial shedding of infectious virus. A key finding from this study was the repeated isolation of infectious virus over time from mucosal swabs including the rectum. This is critically important for the fundamental understanding of both COVID-19 disease and person-to-person transmission.

The SARS-CoV-2-specific immune responses occurred in the second week of infection. The concurrent detection of both IgM and IgG against the receptor binding domain of the spike protein by ELISA coincided with the appearance of a neutralizing antibody response. Mucosally-infected animals had higher ELISA titers than aerosol exposed animals and increased levels of the B cell chemokine CXCL-13 in the plasma during the first week preceding the seroconversion. Plasmablasts peaked in all infected animals on 10 dpi. While a greater magnitude of B cell response seen in mucosal animals could have been secondary to higher inoculum dose, it could also reflect exposure route of which could have implications for optimal immunization. Virus specific T cell responses were not assessed directly. However, proliferation of both CD4 and CD8 T cells, as evidenced by Ki-67 staining, occurred in all animals in the first two weeks of infection. These data are analogous to what occurs in humans with moderate COVID-19 disease [12, 13].

The source of NHP used in pathogenesis and efficacy studies can play an important role in clinical outcome, and thus clear explanation of the source of animals and genetic background for future studies is important. The AGMs used in our study were from the Vervet Research Colony at Wake Forest University. The colony founders were 57 wild-caught animals from St. Kitts in the 1970’s [38, 39]. The animals used here were all male and all ~3.5 years of age (age range is 25–35 years in captivity), making them young and around the age of sexual maturity. Future studies should consider using older animals and/or those directly imported from St. Kitts. Particularly relevant to the COVID-19 pandemic is that AGM from the West Indies spontaneously develop hypertension without dietary intervention or inbreeding [40]. Moreover, they naturally develop type II diabetes and atherosclerosis, making them an excellent species to study pathogenesis of SARS-CoV-2 under conditions representative of the comorbidities associated with severe COVID-19 in humans [41, 42]. Increasing age, genetic variability, and spontaneous hypertension, diabetes, and cardiovascular disease may lead to more severe outcomes in AGMs when infected with SARS-CoV-2, and thus may more accurately reflect the variation seen in the human population.

In summary, we comprehensively evaluated the pathogenesis of SARS-CoV-2 in the AGM model. Our study suggests that AGMs are excellent models for many aspects of COVID-19 in humans: 1) young healthy AGMs may represent subclinical or mild human disease, 2) consistent shedding of infectious virus from respiratory and GI tracts in the absence of overt disease allows experimental understanding of the mechanisms underlying tissue tropism and transmission, and 3) PET/CT imaging modalities are effective at detecting SARS-CoV-2 infection. Critical vaccine trials could use the AGM model to measure virus shedding and/or lung lesions by PET/CT after post-vaccination challenge as measures of vaccine efficacy.

## Methods

### Ethics

The animal work performed adhered to the highest level of humane animal care standards. The University of Pittsburgh is fully accredited by the Association for Assessment and Accreditation of Laboratory Animal Care (AAALAC). All animal work was performed under the standards of the Guide for the Care and Use of Laboratory Animals published by the National Institutes of Health (NIH) and according to the Animal Welfare Act guidelines. All animal studies adhered to the principles stated in the Public Health Services Policy on Humane Care and Use of Laboratory Animals. The University of Pittsburgh Institutional Animal Care and Use Committee (IACUC) approved and oversaw the animal protocols for these studies (#20037014).

### Biological safety

All work with SARS-CoV-2 was conducted under biosafety level-3 (BSL-3) conditions in the University of Pittsburgh Center for Vaccine Research (CVR) and the Regional Biocontainment Laboratory (RBL). Respiratory protection for all personnel when handling infectious samples or working with animals was provided by powered air-purifying respirators (PAPRs; Versaflo TR-300; 3M, St. Paul, MN). Liquid and surface disinfection was performed using Peroxigard disinfectant (1:16 dilution), while solid wastes, caging, and animal wastes were steam sterilized in an autoclave.

### Virology

The SARS-CoV-2 isolate used was a passage 3 (p3) of the Munich isolate described previously [11]. Virus was titrated by plaque assay and titers are expressed as plaque forming units (pfu), and infection was visualized in cells by indirect immunofluorescence, as described [11].

### General animal procedures

Six male AGMs were obtained from the Vervet Research colony at Wake Forest University (S1 Table). All were captive bred animals originally from St. Kitts [38]. They were serologically negative for herpes B virus, SIV, simian T-cell leukemia virus (STLV), and simian retrovirus (SRV). During quarantine, animals underwent telemetry implant surgery, described below. For euthanasia, each animal was sedated with 20 mg/kg ketamine, followed by injection of 200 mg/kg of Beuthanasia IV. Following euthanasia, each animal was perfused via the left ventricle with saline using a variable perfusion machine (GP1000; Fisher Scientific).

### Telemetry surgery and data acquisition

Each AGM was implanted with a DSI PhysioTel Digital radiotelemetry transmitter (DSI Model No. M00) capable of continuously recording body temperature. A subcutaneous pocket was created on the left lateral aspect of the abdomen, and the telemetry implant was placed in the pocket and closed using skin sutures. During acquisition, data was transmitted from the implant to a TRX-1 receiver mounted in the room connected via a Communications Link Controller (CLC) to a computer running Ponemah v6.5 (DSI) software. Pre-exposure data collection began at least seven days in advance of infection. Data collected from Ponemah was exported as 15-minute averages into Excel files which were subsequently analyzed in MatLab 2019a. Using pre-exposure baseline data, an auto-regressive integrated moving average (ARIMA) model was used to forecast body temperature assuming diurnal variation across a 24-hour period. The code is available at https://github.com/ReedLabatPitt/Reed-Lab-Code-Library. Residual temperatures were calculated as actual minus predicted temperatures. Upper and lower limits to determine significant changes were calculated as the product of 3 times the square root of the residual sum of squares from the baseline data.

### Aerosol infection

Aerosol exposures were performed using the Aero3G aerosol management platform as previously described [6]. Jacketed External Telemetry Respiratory Inductive Plethysmography (JET-RIP; DSI) belts were placed around the abdomen and chest of the animal and calibrated to a pneumotach. This allowed monitoring and recording of respiratory function including minute volume during the exposure via the Ponemah v5.4 software platform (DSI). Aerosols were generated using an Aerogen Solo vibrating mesh nebulizer as previously described [43] with a total airflow of 16 l/min into the chamber. Aerosol sampling was performed with an all-glass impinger (AGI) operating at 6 l/min, -6 to -15 psi. Particle size was measured once during each exposure at 5 minutes using an Aerodynamic Particle Sizer (TSI, Shoreview, MN). The median mass aerodynamic diameter (MMAD) across all aerosol infection runs was 1.7 μm. Inhaled dose was calculated based on pre- and post-sampling titers as described [44].

### Multi-route mucosal infection

Virus was administered to two AGM (5 ml of 5x10^5^ pfu/ml) split between 4 sites (nasal (0.5 ml per nare), oral (1 ml), ocular (100 μl, tracheal (2.8 ml)). Intra-tracheal infections were performed using a Wolf bronchoscope; the distal end of the scope was passed between the laryngeal folds into the trachea until the bifurcation of the trachea was visualized. Once the scope was in place, approximately 2.8 ml of virus stock solution was administered into the trachea, followed by 3 ml of sterile saline, and 5 ml of air to flush any remaining fluid from the bronchoscope into the airway.

### Clinical scoring

Clinical signs were recorded twice daily and each animal was given an objective score for each of 3 components. The total score is the sum of all 3 component scores.

Temperature: Normal range (any temp that equals or falls between the upper and lower temperature limits) = 0; significant elevation (above the upper limit) = 1; significant decrease (below the lower limit) = 2; severe hypothermia (<34°C but >31°C) = 3; temperature <31°C = euthanize promptly.

Clinical Appearance/Behavior: normal = 0; lethargic, huddled = 1; piloerection, dehydration, or anorexia = 2; moves only when prodded = 3; no response to prodding = euthanize promptly

Respiratory Signs: none = 0; nasal discharge = 1; increased respiratory rate and effort = 2; respiratory distress (defined as respiratory rate greater than twice the baseline rate) = 3; rales = euthanize promptly.

### Plethysmography

Respiratory function was assessed in anesthetized animals using a head-out plethysmography chamber and pneumotach connected to a digital preamplifier run by Finepointe v2.8 software (DSI). For purposes of this study, we used a universal study modified to collect data similar to the chronic obstructive pulmonary disease (COPD) studies that Finepointe has established for whole-body plethysmography chambers was used. The chamber, pneumotach, and preamplifier were calibrated before use,data was collected for three minutes and analyzed within Finepointe.

### Longitudinal sampling

Animals were sedated for sampling after infection by administration of 10 mg/kg ketamine via intramuscular injection. Once sedated, 2–3 ml of blood was drawn from either the right or left femoral vein into an EDTA tube. Plasma was frozen for serologic, immunologic and virologic assays, and peripheral blood mononuclear cells (PBMCs) were prepared as previously described for flow cytometric studies [6]. CBC analysis was performed using the Abaxis HM5 hematology analyzer. Blood chemistry analysis was performed using the Comprehensive Diagnostic Panel rotor (Abaxis 500–0038) on an Abaxis VS2 chemistry analyzer. Radiographs and mucosal swabs were obtained from each animal. Four types of swabs were obtained at each time point: oral, ocular, nasal, and rectal. Swabs were rotated in place for 10 seconds and then inserted into 1 ml virus transport media (495 ml Opti-MEM and 5 ml of 100 ug/ml Antibiotic/Antimycotic). Each swab was vortexed for 5 seconds, centrifuged to collect the media, and then the media was frozen. For erythrocyte sedimentation rate (ESR), 1 ml of EDTA treated blood was placed into measured capillary tubes for 1 hour. Sedimentation of red blood cells was then recorded in centimeters.

### PBMC thawing and staining

For flow cytometry, PBMC samples were thawed in a 37°C water bath and resuspended in DMEM:F12 media (Fisher 11320–033). Cells were then pelleted (1000 rpm x 5 min) and washed twice using DPBS. Pellets were resuspended in live/dead solution (Fisher L34961) on ice for 20 minutes. Samples were then washed twice with FACS buffer and stained with extracellular antibodies consisting of CD14 (BD 561391), CD16 (BD 562874 and BD561394), CD11b (BD 561887), HLA-DR (BD 339194), CD3 (BD 558124 or BD 557757), CD20 (BD 560734), CD4 (BD 347327), CD8 (BD 335787), NKG2A (Beckman Coulter A60797), CD1c (Biolegend 331506), and CD123 (BD 560826) on ice for 30 min. Stained samples were washed twice with FACS buffer and fixed and permeabilized using BD Cytofix/Cytoperm (BD554714) for 20 min on ice. Samples were washed twice using 1x BD Cytoperm buffer (BD554714) 500 g x 4 min and resuspended in intracellular antibody cocktail consisting of CD38 (BD 560676) and Ki-67 (BD 558616) for 20 minutes on ice. After washing, samples were fixed and inactivated using 4% (w/v) paraformaldehyde, run on a BD LSRII, and analyzed using FlowJo 10.5.0.

### Multiplex cytokine analysis

ProcartaPlex NHP Cytokine & Chemokine Panel 30-plex (Fisher EPX300-400-44-901) was used. For each sample, 25 μl of plasma was used following manufacturer’s instructions. Samples were run on BioRad Bio-Plex 200 reader and results analyzed using Bio-Plex manager software v6.2.

### Radiography

Using a Sedecal Portable X-ray unit with a Ralco X-ray collimator, each sedated animal was placed in ventral dorsal recumbency on top of a double bagged Fugi (25.2 x 30.3 cm or 35.4 x 43.0 cm) cassette. The collimator was focused on the thorax of the animal. The X-ray unit was set to 55 kvp and 2.0 MAS for 0.07 sec. Radiographs were processed using a Med Serv plus digital processor and were interpreted by a board-certified radiologist.

### PET/CT imaging

Animals were sedated with 10 mg/kg ketamine / 0.5 ml atropine before imaging. An intravenous catheter was placed in the saphenous vein and animals were injected with ~ 5 millicurie (mCi) of ^18^F-FDG. An endotracheal tube was placed for ventilation during scanning and the eyes were lubricated with artificial tears. Once placed on the imaging bed, anesthesia was induced with 2.5 to 3% isoflurane which is reduced to 0.8–1.2% for maintenance. Breathing during imaging was maintained using an Inspiration 7i ventilator (eVent Medical, Lake Forest, CA, USA) with the following settings: PF = 9.0 l/min, respiration rate = 18–22 bpm, tidal volume = 60 ml, O2 = 100, PEEP = 5–8 cm H_2_O, peak pressure = 15–18 cm H_2_O, I:E ratio = 1:2.0. A breath hold was conducted during the entirety of the CT acquisition.

PET/CT scans were performed on a MultiScan LFER 150 (Mediso Medical Imaging Systems, Budapest, Hungary). CT acquisition was performed using the following parameters: Semi-circular single field-of-view, 360 projections, 80 kVp, 670 μA, exposure time 90 ms, binning 1:4, voxel size of final image: 500 x 500 μm. PET acquisition was performed 55 min after intravenous injection of ^18^F-FDG with the following parameters: 10 min acquisition, single field-of-view, 1–9 coincidence mode, 5 ns coincidence time window. PET images were reconstructed with the following parameters: Tera-Tomo 3D reconstruction, 400–600 keV energy window, 1–9 coincidence mode, median filter on, spike filter on, voxel size 0.7 mm, 8 iterations, 9 subsets, scatter correction on, attenuation correction based on CT material map segmentation. Serial CT or PET/CT images were acquired pre-infection and at 4 and 11 dpi. Animal A2 was CT scanned at 9 dpi instead of 11 dpi, and animal A1 was scanned at 18 dpi in addition to the standard imaging schedule previously described.

Images were analyzed using OsiriX MD or 64-bit (v.11, Pixmeo, Geneva, Switzerland). Before analysis, PET images were Gaussian smoothed in OsiriX and smoothing was applied to raw data with a 3 x 3 matrix size and a matrix normalization value of 24. Whole lung FDG uptake was measured by first creating a whole lung region-of-interest (ROI) on the lung in the CT scan by creating a 3D growing region highlighting every voxel in the lungs between -1024 and -500 Hounsfield units. This whole lung ROI was copied and pasted to the PET scan and gaps within the ROI were filled in using a closing ROI brush tool with a structuring element radius of 3. All voxels within the lung ROI with a standard uptake value (SUV) below 1.5 were set to zero and the SUVs of the remaining voxels were summed for a total lung FDG uptake (total inflammation) value. Thoracic lymph nodes were analyzed by measuring the maximum SUV within each lymph node using an oval drawing tool. Both total FDG uptake and lymph node uptake values were normalized to back muscle FDG uptake that was measured by drawing cylinder ROIs on the back muscles adjacent to the spine at the same axial level as the carina (SUVCMR; cylinder-muscle-ratio) [18]. PET quantification values were organized in Microsoft Excel and graphed using GraphPad Prism.

### Tissue extraction and processing

For whole tissues, 100 mg of tissue was harvested, suspended in 1.5 ml DPBS (no cations) supplemented with 1% (v/v) fetal bovine serum (FBS) and penicillin-streptomycin [100 iU/100 μg/ml], homogenized using an Omni tissue homogenizer (Omni International). Tissue homogenate or swab eluate (100 μl) was added to 900 μl of Tri-Reagent (ThermoFisher), thoroughly mixed by vortexing. To ensure virus inactivation, the samples were incubated for 10 minutes at room temperature, stored overnight at -80°C prior to removal from the BSL-3 facility. Subsequent storage at -80°C or RNA isolation and one-step qRT-PCR analyses were performed at BSL-2.

### RNA Isolation and one-step qRT-PCR

Tissue and swab RNA was isolated using a standard alcohol precipitation method and eluted in 40 μl of nuclease-free water. For swab samples, 5 μl of polyacryl carrier (Molecular Research Center) was added to the specimen/Tri-Reagent mixture and incubated at room temperature for 30 seconds. The RNA isolation procedure described for tissues was followed for the remainder of the isolation. For quantitation of viral RNA, a multiplex one-step qRT-PCR was performed using the 4x Reliance One-Step Multiplex RT-qPCR Supermix (BioRad), as described [11], using primer and probes targeting the nucleocapsid (N) designed and optimized by the Centers for Disease Control and Prevention (2019-nCoV_N2 forward primer 5’-TTACAAACATTGGCCGCAAA-3’; 2019-nCoV_N2 reverse primer 5’-GCGCGACATTCCGAAGAA-3’ and 2019-nCoV_N2 Probe 5’-FAM-ACAATTTG CCCCCAGCGCTTCAG-BHQ1-3’). 18S rRNA (eukaryotic 18S rRNA endogenous control; Applied Biosystems) was used as an internal control to confirm appropriate specimen collection. The 18S rRNA probes were tagged with a 5’-VIC fluorophore and 3’-TAMRA quencher for multiplexing. Positive-sense vRNA for the standard curve was developed in-house by *in vitro* transcription, using the mMessage mMachine T7 kit (Ambion) and following the manufacturer’s instructions. The limit of detection (LOD) for each one-step qPCR reaction was 23.2 genome copies. The final LOD based on 1 ml or 100 mg of sample was 1,856 genome copies/ml or 100 mg of tissue.

### Serology

Serum neutralizing capacity was determined using an 80% plaque reduction neutralization test (PRNT_80_) as described [11]. Virus-specific total IgG and IgM were measured using ELISAs. ELISA plates (Maxisorp) were coated with 50 ng/well of SARS-CoV-2 RBD (kindly provided by Dr. Seema Lakdawala and prepared according to [45]) diluted in PBS overnight at 4°C. Plates were blocked in 5% (v/v) FBS, 5% (w/v) skim milk in PBS with 0.1% (v/v) Tween-20 for 1 hour at 37°C. Serial dilutions of plasma were made in block and incubated on blocked plates for 2 h at 37°C. Three washes with PBST were performed followed by incubation with goat-anti-monkey IgM(μ)-HRP (Seracare/KPL # 5220–0334) or goat-anti-rhesus IgG (H+L)-HRP (Southern Biotech # 6200–05), both used at a 1:5,000 dilution in blocking solution for 1 hour at 37°C. Three washes with PBST were performed prior to assay development by incubation with TMB (Seracare) for 7 min prior to the addition of TMB stop solution (Seracare). Absorbance values were determined at 450 nm.

### Pathology

Tissues were immersion-fixed in 4% (w/v) paraformaldehyde, routinely processed, cut into 4 μm slides, and stained with hematoxylin and eosin (H&E). Stained slides were interpreted by a board-certified veterinary pathologist.

## Supporting information

S1 Table African green monkey cohort description. (PDF)

S2 Table Markers used for lymphoid and myeloid flow cytometry panels. (PDF)

S1 Fig Clinical parameters in SARS-CoV-2 infected AGM. (A) Total clinical score based on a summation of temperature, appearance/behavior, and respiratory signs (maximum score = 9). (B) Tidal volume measured by plethysmography. (C) whole blood cell count (WBC), (D) lymphocytes, (E) monocytes, (F) neutrophils, (G) hemoglobin, and (H) platelets. Statistical significance determined by ANOVA using GraphPad Prism and indicated by asterisks. Gray shaded area in C-F represent the mean pre-infection level +/- 1 standard deviation. AGM infected by aerosol (closed symbols/solid lines; n = 4) or multi-route mucosal (open symbols/dashed lines; n = 2).
(TIF)

S2 Fig Body temperature changes in SARS-CoV-2-infected AGM. Change in temperature over baseline for indicated animals. (A) A1; (B) A2; (C) A3; (D) A4; (E) M1; (F) M2. Horizontal dashed lines represent the upper and lower limit based on pre-infection data for each animal. Arrows indicate significant elevations.
(TIF)

S3 Fig Blood chemistry parameters during SARS-CoV-2 infection. (A) albumin, (B) blood urea nitrogen, (C) alkaline phosphatase, (D) creatinine, (E) amylase, (F) alanine transaminase, (G) erythrocyte sedimentation rate (ESR). Statistical significance was determined by 2-way ANOVA with multiple comparisons. Asterisks indicate significant changes compared to baseline (time 0) for each animal compared to its own baseline. Gray area in graphs A-F represent the mean of the pre-infection values +/- 1 standard deviation.
(TIF)

S4 Fig Furin cleavage site of SARS-CoV-2 inoculum and swab isolates. Virus were grown from d7 (oral and nasal) and d4 swab (rectal) samples in Vero-E6 cells. RNA was extracted from cell free supernatant virus using Trizol (Invitrogen), cDNA was prepared, and the whole S gene was amplified and sequenced. Chromatograms show the nucleotide sequence coding for the furin-like cleavage signal (RRAR) region of the S protein for each virus isolation. Red asterisks indicate the nucleotide change (double peaks) found in the virus inoculum and the virus isolated on 7 dpi from swabs showing no nucleotide change.
(TIF)

S5 Fig PET/CT images of AGM A1 and A2 infected with SARS-CoV-2. (A) AGM A1 (aerosol). PET/CT scans were obtained pre, 4, 11, and 18 dpi. No abnormal lung tissue at 4 dpi but the LNs were FDG avid. A focal pleural lesion was observed at 11 dpi on the posterior surface of the left upper lobe (FDG+) and an area of opacity in the medial portion of the right lower lobe (FDG-). By 18 dpi, lesions were resolving. Pulmonary infection (yellow arrows); thoracic lymph (green arrows). PET color scale is from 0 to 15 SUV. (B) AGM A2 (aerosol). Only CT scans were obtained at 4 and 9 dpi. On day 4 dpi, A2 had ground glass opacity and thickened vessel structures in the anterior portions of the right upper and right middle lobes and a dense area of disease running vertically through the anterior portion of the right upper lobe. On 9 dpi, some slight opacity was still present in the right middle lobe, but there was no abnormality in the right upper lobe.
(TIF)

S6 Fig PET/CT images of AGM A3 and M2 infected with SARS-CoV-2. (A) AGM A3 (aerosol). A CT scan was obtained at 4 dpi and PET/CT at 11 dpi. At 4 dpi, two foci were visualized in the accessory lobe that were also present at 11 dpi (FDG+). (B) AGM M2 (mucosal). An area of ground glass opacity, a distinct linear-shaped dense lesion in the right lower lobe, and an area of mid-parenchymal opacity in the left upper lobe. All were FDG+ and resolved by 11 dpi. Lymph node FDG uptake was consistent between 4 and 11 dpi. Pulmonary infection (yellow arrows); thoracic lymph nodes (green arrows). PET color scale is from 0–15 SUV.
(TIF)

S7 Fig Mild lung infiltrates in SARS-CoV-2 infected AGM visualized by radiography. Radiographs from AGM M1 (mucosal) at pre, 2, 4, and 7 dpi. Mild non-specific infiltrates were seen in the left lower lobe at 2 and 4 dpi that is resolving by 7 dpi. Radiographs were interpreted by a board-certified radiologist.
(TIF)

S8 Fig Lymphoid Panel Gating Strategy. Representative PBMC sample from 0 dpi. Gating began with collection gate (A), live/dead (B), and singlet inclusion (C). CD14 and CD16 (D) and NKG2A (E) events were excluded from analysis and CD3 expression was evaluated (F). CD3+ events were further separated in CD4 or CD8 (G). CD4+ and CD8+ were then characterized by Ki-67+ (J and H, respectively). CD3- events were evaluated for CD20hi/low HLA-DR+ (I) CD20hiHLA-DR+ and CD20lowHLA-DR+ events were characterized for Ki-67+ (K and L).
(TIF)

S9 Fig Meyloid Panel Gating Strategy. Representative PBMC sample from 0 dpi. Gating began with viable cell gate (A), singlet inclusion (B), and Live/Dead (C) (same graphs as S8 Fig). CD3, CD20 (D) and NKG2A (E) were then excluded from analysis. NKG2A+ cells were evaluated for CD16 expression (F,I). CD3-,CD20-, and NKG2A- cells were then evaluated by CD14 and CD16 (G). CD14+CD16-, CD14+CD16+, and CD14-CD16+ were classified as classical, intermediate, and nonclassical monocytes respectively (G). Classical (H), intermediate (K), and nonclassical (L) were characterized Ki-67+, respectively. CD14- events were evaluated for CD11c (mDC) or CD123 (pDC) (J).
(TIF)

S10 Fig Myeloid and NK cell populations in peripheral blood of SARS-CoV-2 infected AGMs. (A) inflammatory monocytes (CD14+CD16+), (B) Ki-67+ inflammatory monocytes, (C) nonclassical monocytes (CD14-CD16+), (D) Ki-67+ nonclassical monocytes, (E) myeloid dendritic cells (mDCs), and (F) plasmacytoid dendritic cells (pDCs). (G) NK cells, (H) mean fluorescence intensity (MFI) of CD16 expression on NK cells. 2-way ANOVA with multiple comparisons was used to determine statistical significance compared to 0 dpi and is indicated by asterisks above each time point. N.s. in the lower right corner indicates no significant differences.
(TIF)

## References

[1] Woolsey C, Borisevich V, Prasad AN, Agans KN, Deer DJ, Dobias NS, et al Establishment of an African green monkey model for COVID-19. bioRxiv. 2020:2020.05.17.100289. 10.1101/2020.05.17.100289 33235385 PMC7790436

[2] Munster VJ, Feldmann F, Williamson BN, van Doremalen N, Pérez-Pérez L, Schulz J, et al Respiratory disease in rhesus macaques inoculated with SARS-CoV-2. Nature. 2020 10.1038/s41586-020-2324-7 32396922 PMC7486227

[3] Rockx B, Kuiken T, Herfst S, Bestebroer T, Lamers MM, Oude Munnink BB, et al Comparative pathogenesis of COVID-19, MERS, and SARS in a nonhuman primate model. Science. 2020 Epub 2020/04/19. 10.1126/science.abb7314 32303590 PMC7164679

[4] Shan C, Yao Y-F, Yang X-L, Zhou Y-W, Wu J, Gao G, et al Infection with Novel Coronavirus (SARS-CoV-2) Causes Pneumonia in the Rhesus Macaques. Research Square. 2020 10.21203/rs.2.25200/v1 . PMC7364749 32636454

[5] Pandrea I, Apetrei C, Dufour J, Dillon N, Barbercheck J, Metzger M, et al Simian Immunodeficiency Virus SIVagm.sab Infection of Caribbean African Green Monkeys: a New Model for the Study of SIV Pathogenesis in Natural Hosts. Journal of Virology. 2006;80(10):4858 10.1128/JVI.80.10.4858-4867.2006 16641277 PMC1472068

[6] Wonderlich ER, Caroline AL, McMillen CM, Walters AW, Reed DS, Barratt-Boyes SM, et al Peripheral Blood Biomarkers of Disease Outcome in a Monkey Model of Rift Valley Fever Encephalitis. Journal of Virology. 2018;92(3):e01662–17. 10.1128/JVI.01662-17 29118127 PMC5774883

[7] Layton RC, Mega W, McDonald JD, Brasel TL, Barr EB, Gigliotti AP, et al Levofloxacin cures experimental pneumonic plague in African green monkeys. PLoS Negl Trop Dis. 2011;5(2):e959 Epub 2011/02/25. 10.1371/journal.pntd.0000959 21347450 PMC3035670

[8] Johnston SC, Briese T, Bell TM, Pratt WD, Shamblin JD, Esham HL, et al Detailed analysis of the African green monkey model of Nipah virus disease. PLoS One. 2015;10(2):e0117817 Epub 2015/02/24. 10.1371/journal.pone.0117817 25706617 PMC4338303

[9] Durbin AP, Elkins WR, Murphy BR. African green monkeys provide a useful nonhuman primate model for the study of human parainfluenza virus types-1, -2, and -3 infection. Vaccine. 2000;18(22):2462–9. Epub 2000/03/30. 10.1016/s0264-410x(99)00575-7 . 10738104

[10] McAuliffe J, Vogel L, Roberts A, Fahle G, Fischer S, Shieh WJ, et al Replication of SARS coronavirus administered into the respiratory tract of African Green, rhesus and cynomolgus monkeys. Virology. 2004;330(1):8–15. Epub 2004/11/06. 10.1016/j.virol.2004.09.030 15527829 PMC7111808

[11] Klimstra WB, Tilston-Lunel NL, Nambulli S, Boslett J, McMillen CM, Gilliland T, et al SARS-CoV-2 growth, furin-cleavage-site adaptation and neutralization using serum from acutely infected, hospitalized COVID-19 patients. Journal of General Virology. 2020;In press. 10.1099/jgv.0.001481 32821033 PMC7879561

[12] Kuri-Cervantes L, Pampena MB, Meng W, Rosenfeld AM, Ittner CAG, Weisman AR, et al Immunologic perturbations in severe COVID-19/SARS-CoV-2 infection. bioRxiv. 2020:2020.05.18.101717. 10.1101/2020.05.18.101717 32511394 PMC7263541

[13] Mathew D, Giles JR, Baxter AE, Greenplate AR, Wu JE, Alanio C, et al Deep immune profiling of COVID-19 patients reveals patient heterogeneity and distinct immunotypes with implications for therapeutic interventions. bioRxiv. 2020:2020.05.20.106401. 10.1101/2020.05.20.106401 32669297 PMC7402624

[14] Lin PL, Coleman T, Carney JP, Lopresti BJ, Tomko J, Fillmore D, et al Radiologic Responses in Cynomolgus Macaques for Assessing Tuberculosis Chemotherapy Regimens. Antimicrob Agents Chemother. 2013;57(9):4237–44. Epub 2013/06/26. 10.1128/AAC.00277-13 23796926 PMC3754323

[15] Lin PL, Ford CB, Coleman MT, Myers AJ, Gawande R, Ioerger T, et al Sterilization of granulomas is common in active and latent tuberculosis despite within-host variability in bacterial killing. Nat Med. 2014;20(1):75–9. Epub 2013/12/18. 10.1038/nm.3412 24336248 PMC3947310

[16] Cadena AM, Klein EC, White AG, Tomko JA, Chedrick CL, Reed DS, et al Very Low Doses of Mycobacterium tuberculosis Yield Diverse Host Outcomes in Common Marmosets (Callithrix jacchus). Comp Med. 2016;66(5):412–9. Epub 2016/10/26. 27780009 PMC5073067

[17] Martin CJ, Cadena AM, Leung VW, Lin PL, Maiello P, Hicks N, et al Digitally Barcoding Mycobacterium tuberculosis Reveals In Vivo Infection Dynamics in the Macaque Model of Tuberculosis. mBio. 2017;8(3). Epub 2017/05/11. 10.1128/mBio.00312-17 28487426 PMC5424202

[18] White AG, Maiello P, Coleman MT, Tomko JA, Frye LJ, Scanga CA, et al Analysis of 18FDG PET/CT Imaging as a Tool for Studying Mycobacterium tuberculosis Infection and Treatment in Non-human Primates. J Vis Exp. 2017;(127). Epub 2017/09/21. 10.3791/56375 28930979 PMC5752181

[19] Maiello P, DiFazio RM, Cadena AM, Rodgers MA, Lin PL, Scanga CA, et al Rhesus Macaques Are More Susceptible to Progressive Tuberculosis than Cynomolgus Macaques: a Quantitative Comparison. Infect Immun. 2018;86(2). Epub 2017/09/28. 10.1128/IAI.00505-17 28947646 PMC5778369

[20] Long QX, Liu BZ, Deng HJ, Wu GC, Deng K, Chen YK, et al Antibody responses to SARS-CoV-2 in patients with COVID-19. Nat Med. 2020 Epub 2020/05/01. 10.1038/s41591-020-0897-1 . 32350462

[21] Xiang F, Wang X, He X, Peng Z, Yang B, Zhang J, et al Antibody Detection and Dynamic Characteristics in Patients with COVID-19. Clin Infect Dis. 2020 Epub 2020/04/20. 10.1093/cid/ciaa461 32306047 PMC7188146

[22] Suhandynata RT, Hoffman MA, Kelner MJ, McLawhon RW, Reed SL, Fitzgerald RL. Longitudinal Monitoring of SARS-CoV-2 IgM and IgG Seropositivity to Detect COVID-19. J Appl Lab Med. 2020 Epub 2020/05/20. 10.1093/jalm/jfaa079 . 32428207 PMC7313967

[23] Xu X, Sun J, Nie S, Li H, Kong Y, Liang M, et al Seroprevalence of immunoglobulin M and G antibodies against SARS-CoV-2 in China. Nature Medicine. 2020 10.1038/s41591-020-0949-6 32504052

[24] Freeman MC, Peek CT, Becker MM, Smith EC, Denison MR. Coronaviruses Induce Entry-Independent, Continuous Macropinocytosis. mBio. 2014;5(4):e01340–14. 10.1128/mBio.01340-14 25096879 PMC4128357

[25] Xu Z, Shi L, Wang Y, Zhang J, Huang L, Zhang C, et al Pathological findings of COVID-19 associated with acute respiratory distress syndrome. The Lancet Respiratory Medicine. 2020;8(4):420–2. 10.1016/S2213-2600(20)30076-X 32085846 PMC7164771

[26] Smits SL, van den Brand JM, de Lang A, Leijten LM, van Ijcken WF, van Amerongen G, et al Distinct severe acute respiratory syndrome coronavirus-induced acute lung injury pathways in two different nonhuman primate species. J Virol. 2011;85(9):4234–45. Epub 2011/02/18. 10.1128/JVI.02395-10 21325418 PMC3126247

[27] Kim GU, Kim MJ, Ra SH, Lee J, Bae S, Jung J, et al Clinical characteristics of asymptomatic and symptomatic patients with mild COVID-19. Clin Microbiol Infect. 2020 Epub 2020/05/04. 10.1016/j.cmi.2020.04.040 32360780 PMC7252018

[28] Huang L, Zhang X, Zhang X, Wei Z, Zhang L, Xu J, et al Rapid asymptomatic transmission of COVID-19 during the incubation period demonstrating strong infectivity in a cluster of youngsters aged 16–23 years outside Wuhan and characteristics of young patients with COVID-19: A prospective contact-tracing study. J Infect. 2020;80(6):e1–e13. Epub 2020/04/14. 10.1016/j.jinf.2020.03.006 32283156 PMC7194554

[29] Polverari G, Arena V, Ceci F, Pelosi E, Ianniello A, Poli E, et al 18F-Fluorodeoxyglucose Uptake in Patient With Asymptomatic Severe Acute Respiratory Syndrome Coronavirus 2 (Coronavirus Disease 2019) Referred to Positron Emission Tomography/Computed Tomography for NSCLC Restaging. Journal of Thoracic Oncology. 2020;15(6):1078–80. 10.1016/j.jtho.2020.03.022 32243920 PMC7270857

[30] Tulchinsky M, Fotos JS, Slonimsky E. Incidental CT Findings Suspicious for COVID-19-Associated Pneumonia on Nuclear Medicine Examinations: Recognition and Management Plan. Clin Nucl Med. 2020;45(7):531–3. Epub 2020/06/06. 10.1097/RLU.0000000000003100 32502091 PMC7217125

[31] Lütje S, Marinova M, Kütting D, Attenberger U, Essler M, Bundschuh RA. Nuclear medicine in SARS-CoV-2 pandemia: 18F-FDG-PET/CT to visualize COVID-19. Nuklearmedizin. 2020;59(3):276–80. Epub 2020/04/08. 10.1055/a-1152-2341 . 32259853

[32] Finch CL, Crozier I, Lee JH, Byrum R, Cooper TK, Liang J, et al Characteristic and quantifiable COVID-19-like abnormalities in CT- and PET/CT-imaged lungs of SARS-CoV-2-infected crab-eating macaques (&lt;em&gt;Macaca fascicularis&lt;/em&gt;). bioRxiv. 2020:2020.05.14.096727. 10.1101/2020.05.14.096727 32511338 PMC7241101

[33] Riley S, Fraser C, Donnelly CA, Ghani AC, Abu-Raddad LJ, Hedley AJ, et al Transmission dynamics of the etiological agent of SARS in Hong Kong: impact of public health interventions. Science. 2003;300(5627):1961–6. Epub 2003/05/27. 10.1126/science.1086478 . 12766206

[34] McKinney KR, Gong YY, Lewis TG. Environmental transmission of SARS at Amoy Gardens. J Environ Health. 2006;68(9):26–30; quiz 51–2. Epub 2006/05/16. . 16696450

[35] Chen C, Gao G, Xu Y, Pu L, Wang Q, Wang L, et al SARS-CoV-2-Positive Sputum and Feces After Conversion of Pharyngeal Samples in Patients With COVID-19. Ann Intern Med. 2020 Epub 2020/04/01. 10.7326/M20-0991 32227141 PMC7133055

[36] Chen Y, Chen L, Deng Q, Zhang G, Wu K, Ni L, et al The presence of SARS-CoV-2 RNA in the feces of COVID-19 patients. J Med Virol. 2020 Epub 2020/04/04. 10.1002/jmv.25825 . 32243607

[37] Wang W, Xu Y, Gao R, Lu R, Han K, Wu G, et al Detection of SARS-CoV-2 in Different Types of Clinical Specimens. JAMA. 2020 Epub 2020/03/12. 10.1001/jama.2020.3786 32159775 PMC7066521

[38] Jasinska AJ, Schmitt CA, Service SK, Cantor RM, Dewar K, Jentsch JD, et al Systems Biology of the Vervet Monkey. ILAR Journal. 2013;54(2):122–43. 10.1093/ilar/ilt049 24174437 PMC3814400

[39] Fairbanks LA, Newman TK, Bailey JN, Jorgensen MJ, Breidenthal SE, Ophoff RA, et al Genetic contributions to social impulsivity and aggressiveness in vervet monkeys. Biological Psychiatry. 2004;55(6):642–7. 10.1016/j.biopsych.2003.12.005 15013834

[40] Rhoads MK, Goleva SB, Beierwaltes WH, Osborn JL. Renal vascular and glomerular pathologies associated with spontaneous hypertension in the nonhuman primate Chlorocebus aethiops sabaeus. American Journal of Physiology-Regulatory, Integrative and Comparative Physiology. 2017;313(3):R211–R8. 10.1152/ajpregu.00026.2017 28659284

[41] Guan WJ, Liang WH, Zhao Y, Liang HR, Chen ZS, Li YM, et al Comorbidity and its impact on 1590 patients with COVID-19 in China: a nationwide analysis. Eur Respir J. 2020;55(5). Epub 2020/03/29. 10.1183/13993003.00547–2020 32217650 PMC7098485

[42] Du RH, Liang LR, Yang CQ, Wang W, Cao TZ, Li M, et al Predictors of mortality for patients with COVID-19 pneumonia caused by SARS-CoV-2: a prospective cohort study. Eur Respir J. 2020;55(5). Epub 2020/04/10. 10.1183/13993003.00524–2020 32269088 PMC7144257

[43] Bowling JD, O’Malley KJ, Klimstra WB, Hartman AL, Reed DS. A Vibrating Mesh Nebulizer as an Alternative to the Collison Three-Jet Nebulizer for Infectious Disease Aerobiology. Applied and Environmental Microbiology. 2019;85(17):e00747–19. 10.1128/AEM.00747-19 31253680 PMC6696971

[44] Roy CJ, Pitt LM. Infectious Disease Aerobiology: Aerosol Challenge Methods In: Swearengen JR, editor. Biodefense: Research Methodology and Animal Models. Boca Raton, FL: CRC Press; 2005 p. 61–76.

[45] Stadlbauer D, Amanat F, Chromikova V, Jiang K, Strohmeier S, Arunkumar GA, et al SARS-CoV-2 Seroconversion in Humans: A Detailed Protocol for a Serological Assay, Antigen Production, and Test Setup. Curr Protoc Microbiol. 2020;57(1):e100 Epub 2020/04/18. 10.1002/cpmc.100 32302069 PMC7235504

