# Supplementary material for: SARS-CoV-2 infection of African green monkeys results in mild respiratory disease discernible by PET/CT imaging and shedding of infectious virus from both respiratory and gastrointestinal tracts
Source: PLoS Pathog. 2020 Sep 18;16(9):e1008903. doi: 10.1371/journal.ppat.1008903 (PMC7535860; doi:10.1371/journal.ppat.1008903)
Supplement: S1 Table — (PDF) [file ppat.1008903.s001.pdf]

S1 Table. African green monkey cohort description

Animal ID	Sex	Age (years)	Weight (kg)	Infection Route	Exposure dose (log_{10} pfu)	Euthanasia dpi	Outcome
A1	M	3.7	5.3	Aero	4.2	35	mild disease
A2	M	3.6	6.3	Aero	4.0	35	mild disease
A3	M	3.6	4.9	Aero	4.2	28	mild disease; infection at telemetry implant site
A4	M	3.5	4.4	Aero	3.7	28	mild disease
M1	M	3.3	4.2	Multi-route mucosal	6.4	28	mild disease
M2	M	3.2	4.3	Multi-route mucosal	6.4	28	mild disease
