# Supplementary material for: SARS-CoV-2 infection of African green monkeys results in mild respiratory disease discernible by PET/CT imaging and shedding of infectious virus from both respiratory and gastrointestinal tracts
Source: PLoS Pathog. 2020 Sep 18;16(9):e1008903. doi: 10.1371/journal.ppat.1008903 (PMC7535860; doi:10.1371/journal.ppat.1008903)
Supplement: S2 Table — (PDF) [file ppat.1008903.s002.pdf]

S2 Table: Markers used for lymphoid and myeloid flow cytometry panels

Lymphoid	Myeloid
CD14, CD16 (dump)	CD3, CD20, NKG2A (dump)
Live/dead	Live/dead
CD3	CD14
CD4	CD16
CD8	CD11c
HLA-DR	HLA-DR
NKG2A	CD123
CD38	CD38
CD27	Ki-67
CD20	
Ki-67	
