# Supplementary figures and images for: SARS-CoV-2 infection of African green monkeys results in mild respiratory disease discernible by PET/CT imaging and shedding of infectious virus from both respiratory and gastrointestinal tracts
Source: PLoS Pathog. 2020 Sep 18;16(9):e1008903. doi: 10.1371/journal.ppat.1008903 (PMC7535860; doi:10.1371/journal.ppat.1008903)

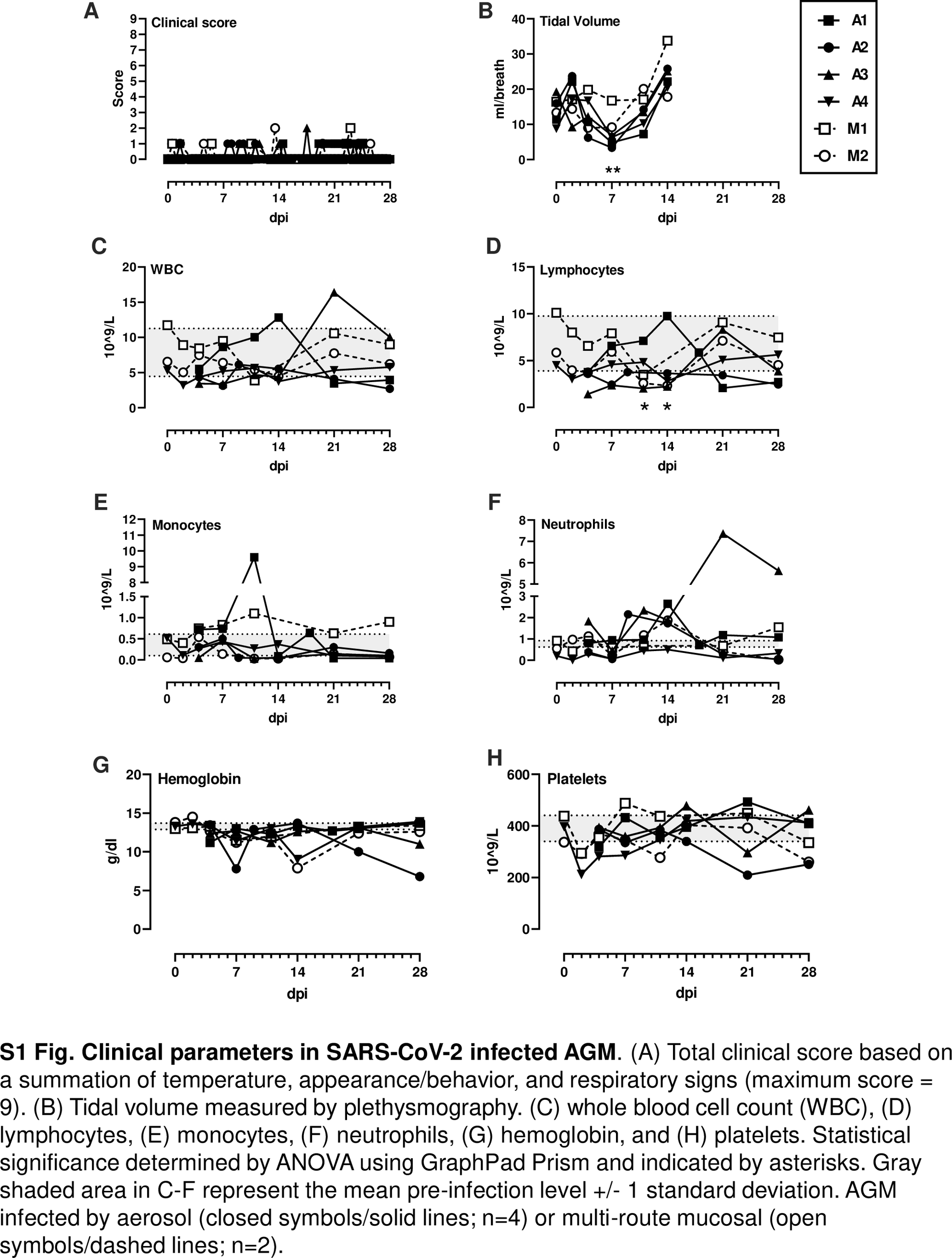

Supplement: S1 Fig — (A) Total clinical score based on a summation of temperature, appearance/behavior, and respiratory signs (maximum score = 9). (B) Tidal volume measured by plethysmography. (C) whole blood cell count (WBC), (D) lymphocytes, (E) monocytes, (F) neutrophils, (G) hemoglobin, and (H) platelets. Statistical significance determined by ANOVA using GraphPad Prism and indicated by asterisks. Gray shaded area in C-F represent the mean pre-infection level +/- 1 standard deviation. AGM infected by aerosol (closed symbols/solid lines; n = 4) or multi-route mucosal (open symbols/dashed lines; n = 2). (TIF) [file ppat.1008903.s003.tif]

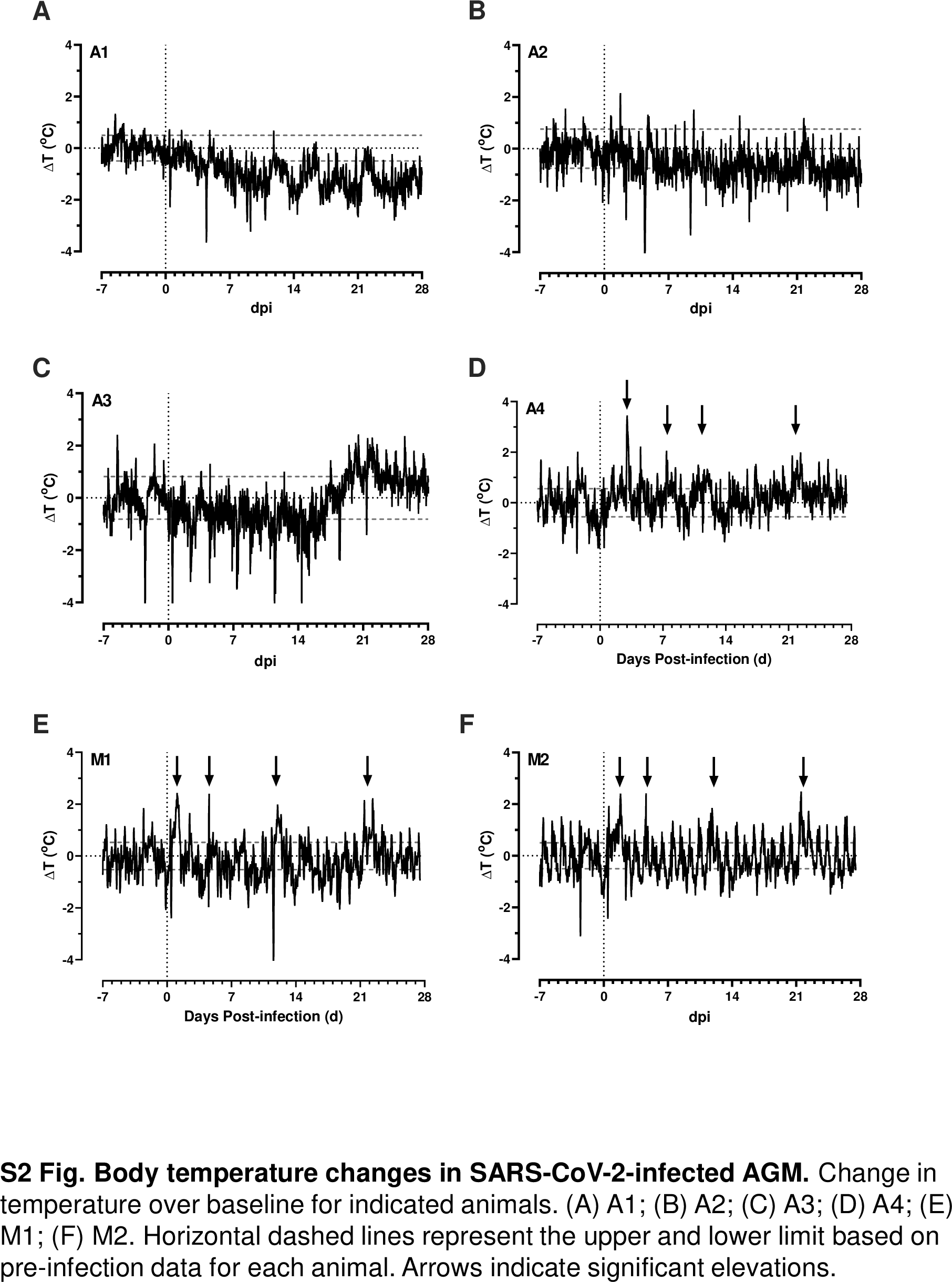

Supplement: S2 Fig — Change in temperature over baseline for indicated animals. (A) A1; (B) A2; (C) A3; (D) A4; (E) M1; (F) M2. Horizontal dashed lines represent the upper and lower limit based on pre-infection data for each animal. Arrows indicate significant elevations. (TIF) [file ppat.1008903.s004.tif]

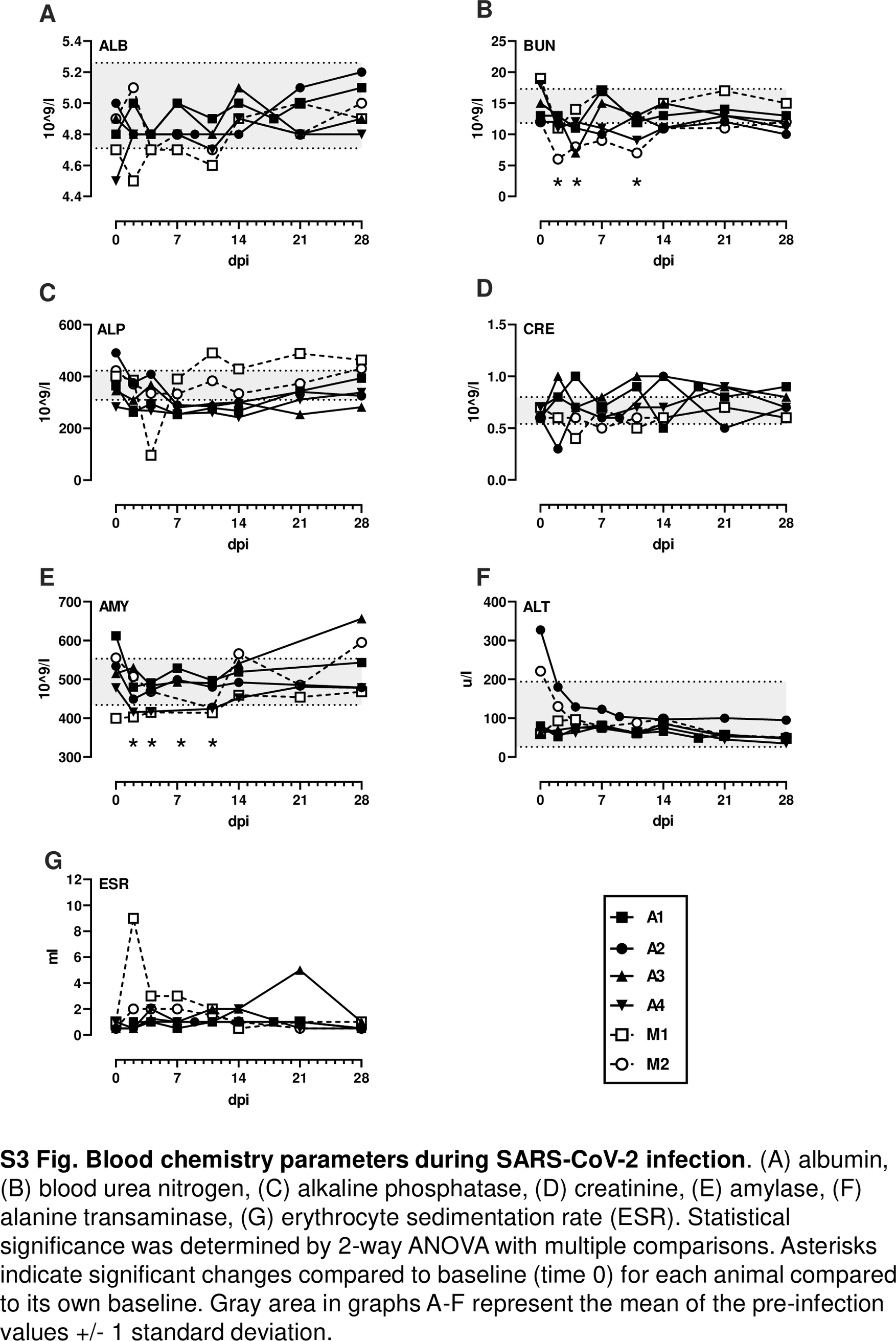

Supplement: S3 Fig — (A) albumin, (B) blood urea nitrogen, (C) alkaline phosphatase, (D) creatinine, (E) amylase, (F) alanine transaminase, (G) erythrocyte sedimentation rate (ESR). Statistical significance was determined by 2-way ANOVA with multiple comparisons. Asterisks indicate significant changes compared to baseline (time 0) for each animal compared to its own baseline. Gray area in graphs A-F represent the mean of the pre-infection values +/- 1 standard deviation. (TIF) [file ppat.1008903.s005.tif]

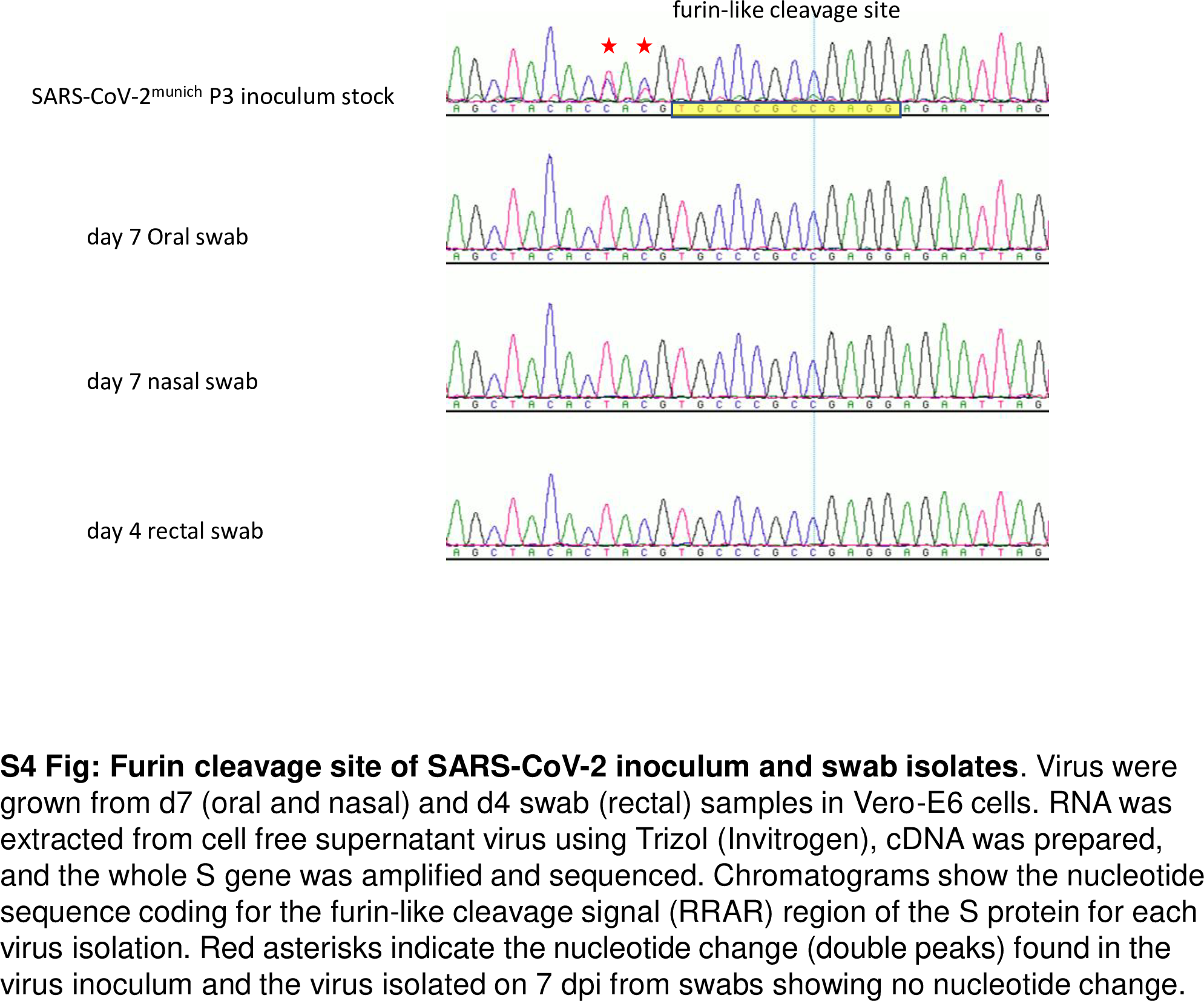

Supplement: S4 Fig — Virus were grown from d7 (oral and nasal) and d4 swab (rectal) samples in Vero-E6 cells. RNA was extracted from cell free supernatant virus using Trizol (Invitrogen), cDNA was prepared, and the whole S gene was amplified and sequenced. Chromatograms show the nucleotide sequence coding for the furin-like cleavage signal (RRAR) region of the S protein for each virus isolation. Red asterisks indicate the nucleotide change (double peaks) found in the virus inoculum and the virus isolated on 7 dpi from swabs showing no nucleotide change. (TIF) [file ppat.1008903.s006.tif]

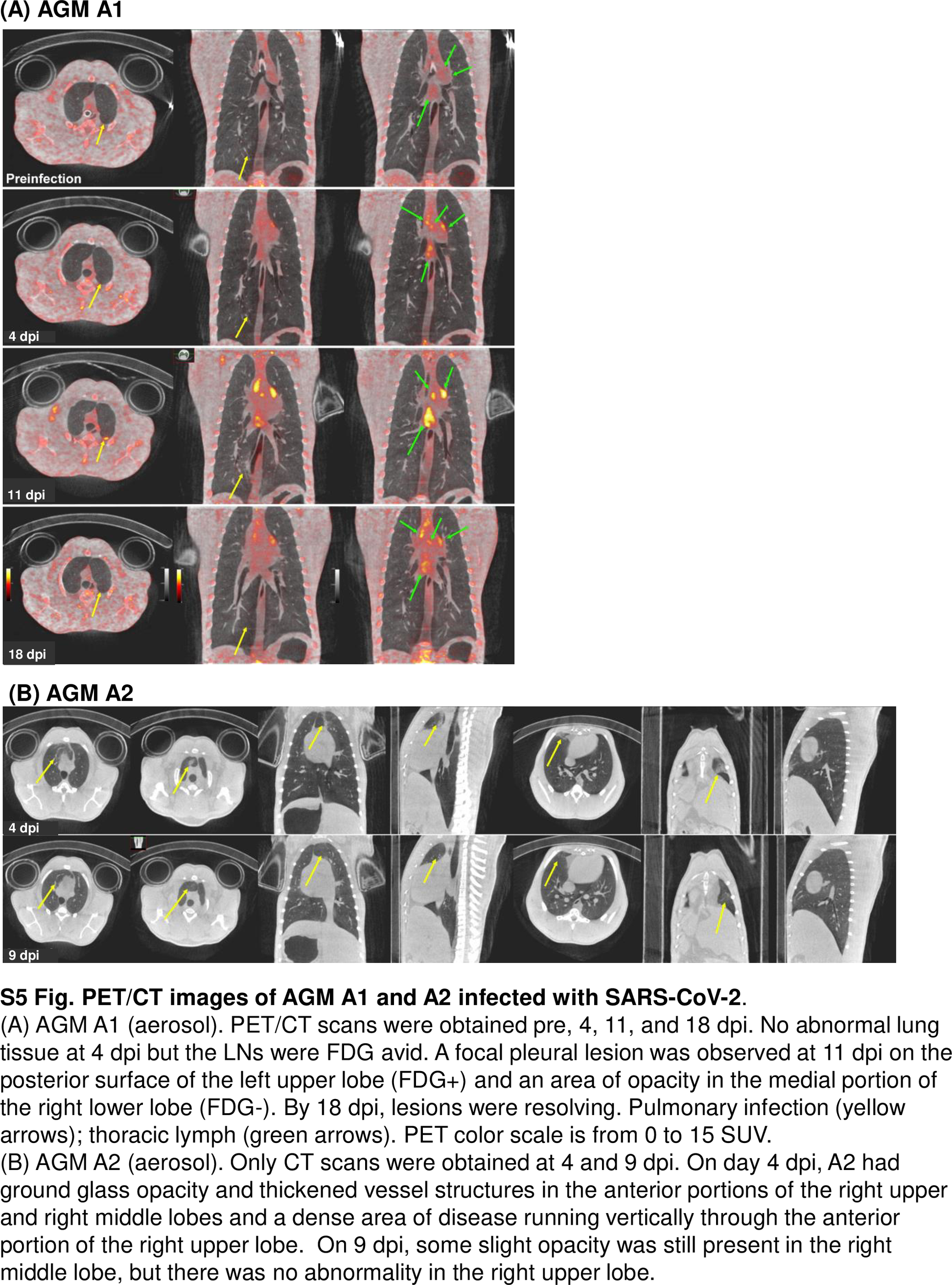

Supplement: S5 Fig — (A) AGM A1 (aerosol). PET/CT scans were obtained pre, 4, 11, and 18 dpi. No abnormal lung tissue at 4 dpi but the LNs were FDG avid. A focal pleural lesion was observed at 11 dpi on the posterior surface of the left upper lobe (FDG+) and an area of opacity in the medial portion of the right lower lobe (FDG-). By 18 dpi, lesions were resolving. Pulmonary infection (yellow arrows); thoracic lymph (green arrows). PET color scale is from 0 to 15 SUV. (B) AGM A2 (aerosol). Only CT scans were obtained at 4 and 9 dpi. On day 4 dpi, A2 had ground glass opacity and thickened vessel structures in the anterior portions of the right upper and right middle lobes and a dense area of disease running vertically through the anterior portion of the right upper lobe. On 9 dpi, some slight opacity was still present in the right middle lobe, but there was no abnormality in the right upper lobe. (TIF) [file ppat.1008903.s007.tif]

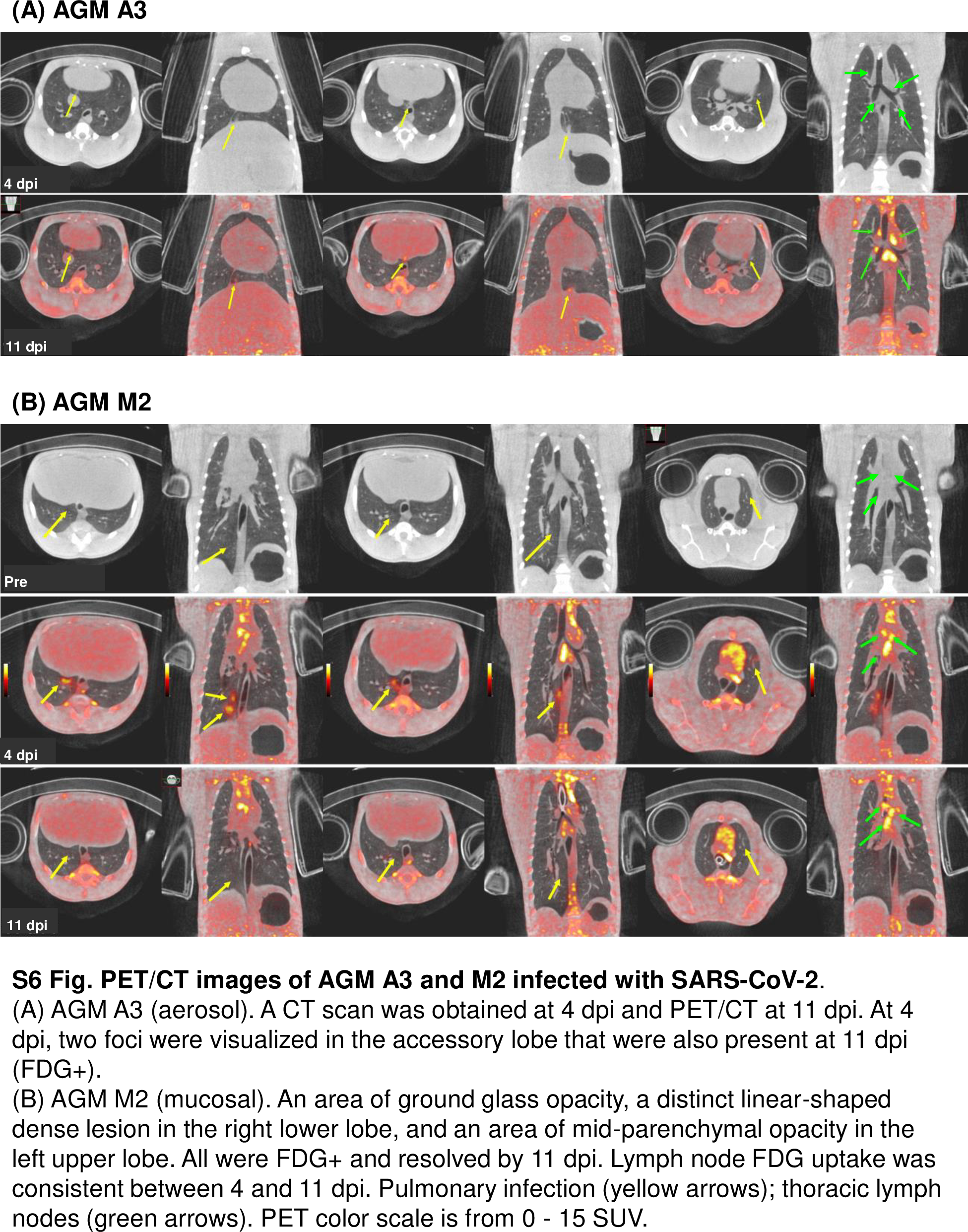

Supplement: S6 Fig — (A) AGM A3 (aerosol). A CT scan was obtained at 4 dpi and PET/CT at 11 dpi. At 4 dpi, two foci were visualized in the accessory lobe that were also present at 11 dpi (FDG+). (B) AGM M2 (mucosal). An area of ground glass opacity, a distinct linear-shaped dense lesion in the right lower lobe, and an area of mid-parenchymal opacity in the left upper lobe. All were FDG+ and resolved by 11 dpi. Lymph node FDG uptake was consistent between 4 and 11 dpi. Pulmonary infection (yellow arrows); thoracic lymph nodes (green arrows). PET color scale is from 0–15 SUV. (TIF) [file ppat.1008903.s008.tif]

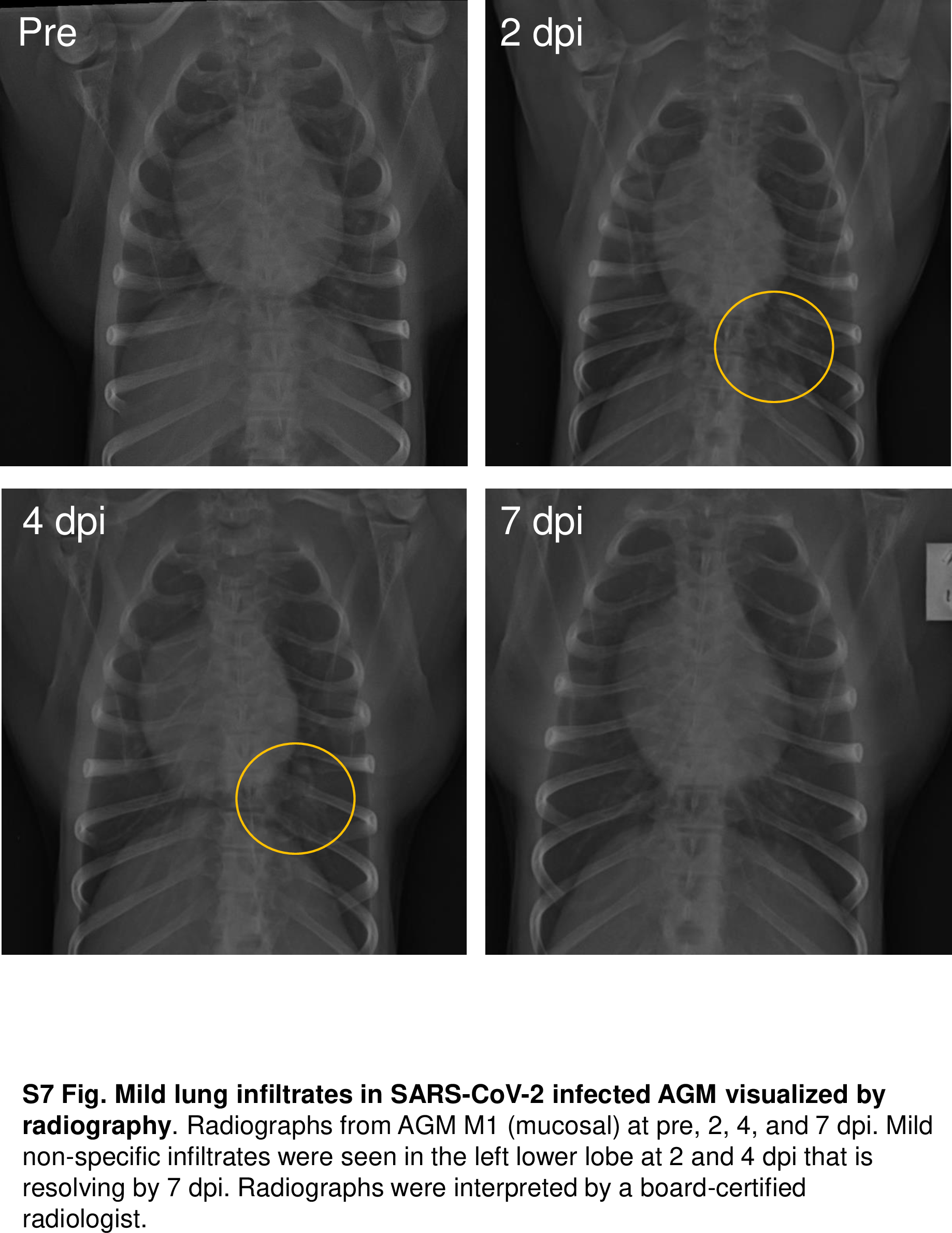

Supplement: S7 Fig — Radiographs from AGM M1 (mucosal) at pre, 2, 4, and 7 dpi. Mild non-specific infiltrates were seen in the left lower lobe at 2 and 4 dpi that is resolving by 7 dpi. Radiographs were interpreted by a board-certified radiologist. (TIF) [file ppat.1008903.s009.tif]

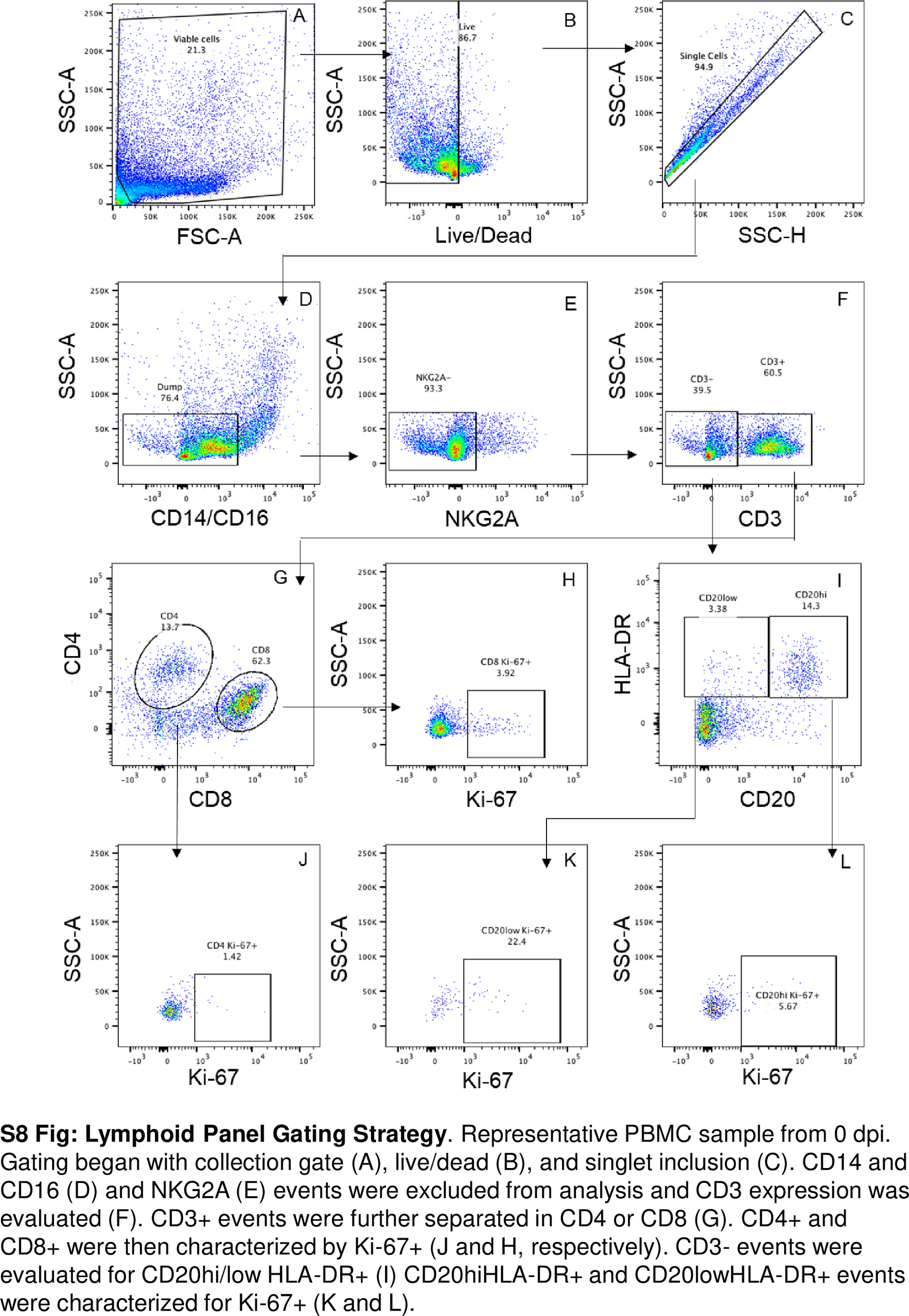

Supplement: S8 Fig — Representative PBMC sample from 0 dpi. Gating began with collection gate (A), live/dead (B), and singlet inclusion (C). CD14 and CD16 (D) and NKG2A (E) events were excluded from analysis and CD3 expression was evaluated (F). CD3+ events were further separated in CD4 or CD8 (G). CD4+ and CD8+ were then characterized by Ki-67+ (J and H, respectively). CD3- events were evaluated for CD20hi/low HLA-DR+ (I) CD20hiHLA-DR+ and CD20lowHLA-DR+ events were characterized for Ki-67+ (K and L). (TIF) [file ppat.1008903.s010.tif]

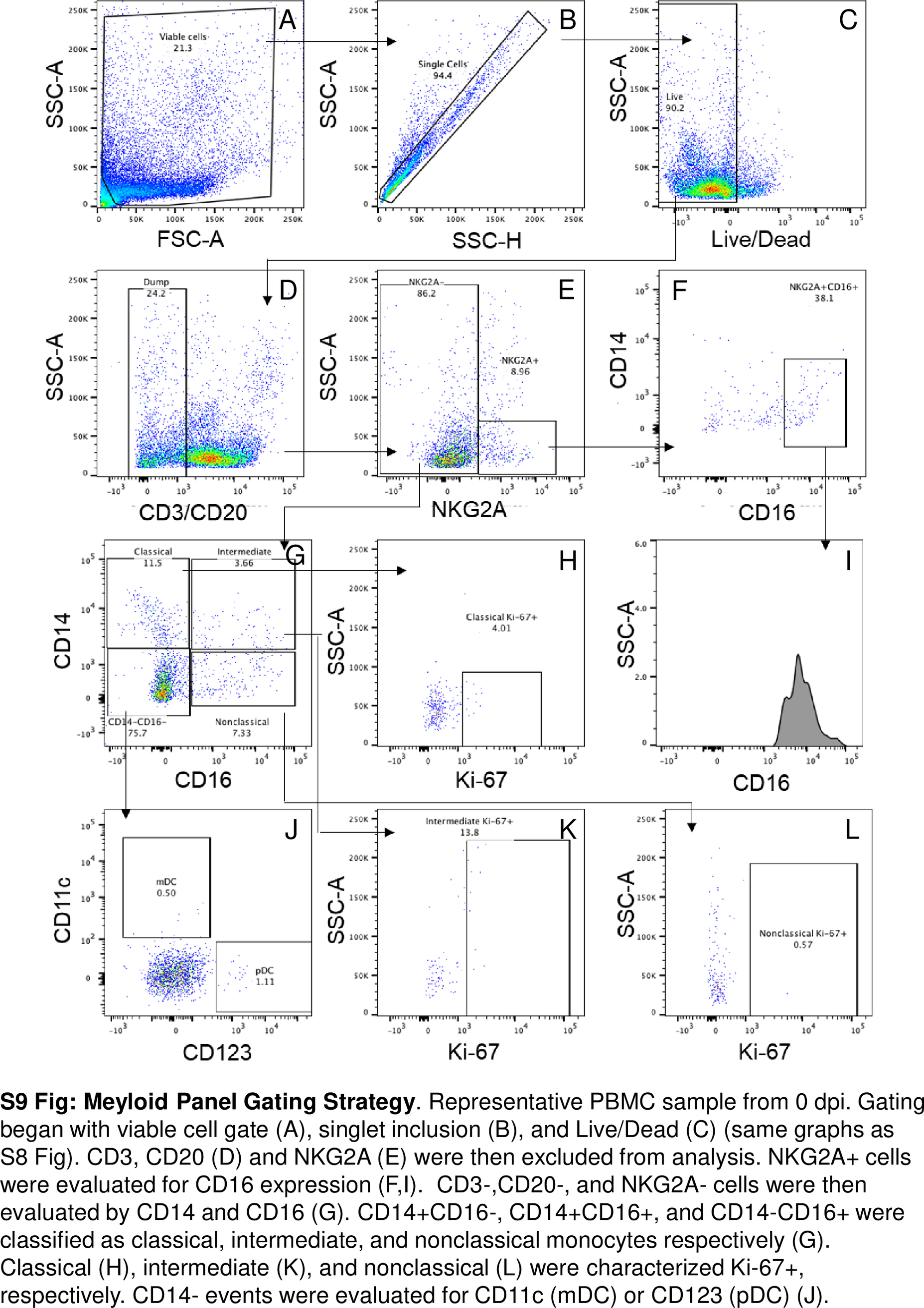

Supplement: S9 Fig — Representative PBMC sample from 0 dpi. Gating began with viable cell gate (A), singlet inclusion (B), and Live/Dead (C) (same graphs as S8 Fig). CD3, CD20 (D) and NKG2A (E) were then excluded from analysis. NKG2A+ cells were evaluated for CD16 expression (F,I). CD3-,CD20-, and NKG2A- cells were then evaluated by CD14 and CD16 (G). CD14+CD16-, CD14+CD16+, and CD14-CD16+ were classified as classical, intermediate, and nonclassical monocytes respectively (G). Classical (H), intermediate (K), and nonclassical (L) were characterized Ki-67+, respectively. CD14- events were evaluated for CD11c (mDC) or CD123 (pDC) (J). (TIF) [file ppat.1008903.s011.tif]

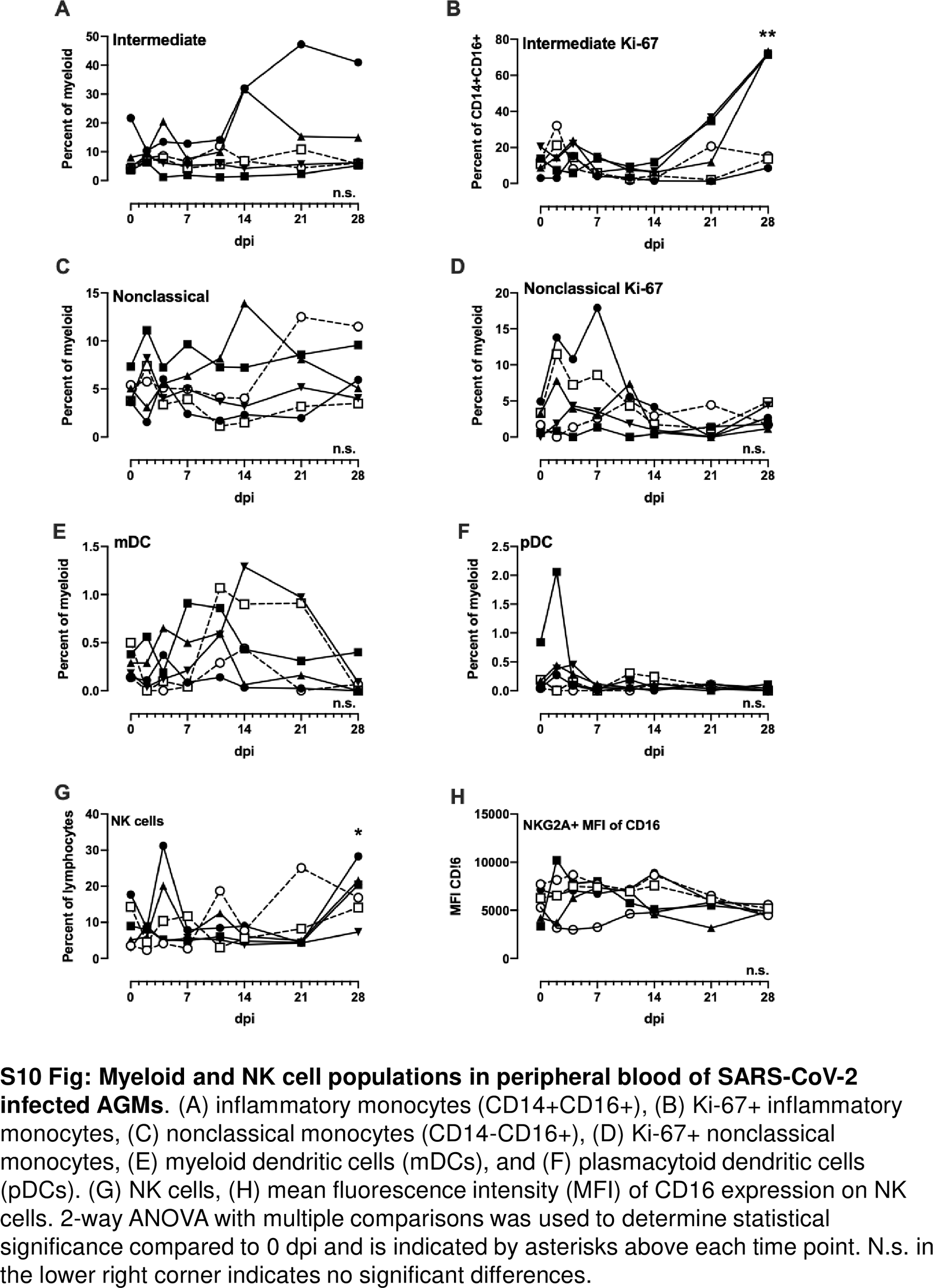

Supplement: S10 Fig — (A) inflammatory monocytes (CD14+CD16+), (B) Ki-67+ inflammatory monocytes, (C) nonclassical monocytes (CD14-CD16+), (D) Ki-67+ nonclassical monocytes, (E) myeloid dendritic cells (mDCs), and (F) plasmacytoid dendritic cells (pDCs). (G) NK cells, (H) mean fluorescence intensity (MFI) of CD16 expression on NK cells. 2-way ANOVA with multiple comparisons was used to determine statistical significance compared to 0 dpi and is indicated by asterisks above each time point. N.s. in the lower right corner indicates no significant differences. (TIF) [file ppat.1008903.s012.tif]
